# Super-enhancers and efficacy of triptolide in small cell carcinoma of the ovary hypercalcemic type

**DOI:** 10.1016/j.isci.2025.111770

**Published:** 2025-01-07

**Authors:** Jessica D. Lang, William Selleck, Shawn Striker, Nicolle A. Hipschman, Rochelle Kofman, Anthony N. Karnezis, Felix K.F. Kommoss, Friedrich Kommoss, Jae Rim Wendt, Salvatore J. Facista, William P.D. Hendricks, Krystal A. Orlando, Patrick Pirrotte, Elizabeth A. Raupach, Victoria L. Zismann, Yemin Wang, David G. Huntsman, Bernard E. Weissman, Jeffrey M. Trent

**Affiliations:** 1 Division of Integrated Cancer Genomics, Translational Genomics Research Institute (TGen), Phoenix, AZ 85004, USA; 2 Department of Pathology and Laboratory Medicine, UW Carbone Cancer Center, and Center for Human Genomics and Precision Medicine, University of Wisconsin-Madison, Madison, WI 53705, USA; 3 Department of Pathology and Laboratory Medicine, University of California Davis, Sacramento, CA 95817, USA; 4 Institute of Pathology, University Hospital Heidelberg, 69120 Heidelberg, Germany; 5 Institute of Pathology, Medizin Campus Bodensee, 88048 Friedrichshafen, Germany; 6 Department of Pathology and Laboratory Medicine, and the Lineberger Comprehensive Cancer Center, University of North Carolina, Chapel Hill, NC 27599, USA; 7 Collaborative Center for Translational Mass Spectrometry, Translational Genomics Research Institute (TGen), Phoenix, AZ 85004, USA; 8 Department of Pathology and Laboratory Medicine, University of British Columbia, Vancouver, BC V6T 1Z7, Canada; 9 Canada and Department of Molecular Oncology, British Columbia Cancer Research Centre, Vancouver, BC V5Z 0B4, Canada; 10 Department of Obstetrics and Gynaecology, University of British Columbia, Vancouver, BC V6T 1Z3, Canada

**Keywords:** Molecular biology, Complex system biology, Cancer

## Abstract

Small cell carcinoma of the ovary-hypercalcemic type (SCCOHT) is a rare ovarian cancer affecting young females and is driven by the loss of both SWI/SNF ATPases SMARCA4 and SMARCA2. As loss of SWI/SNF alters enhancers, we hypothesized that super-enhancers, which regulate oncogene expression in cancer, are disparately impacted by SWI/SNF loss. We discovered differences between SWI/SNF occupancy at enhancers vs. super-enhancers. SCCOHT super-enhancer target genes were enriched in developmental processes, most notably nervous system development. This may further support neuronal cell-of-origin previously proposed. We found high sensitivity of SCCOHT cell lines to triptolide. Triptolide inhibits expression of many super-enhancer-associated genes, including oncogenes. *SALL4* expression is decreased by triptolide and is highly expressed in SCCOHT tumors. In patient-derived xenograft models, triptolide and prodrug minnelide effectively inhibit tumor growth. These results reveal unique features of super-enhancers in SCCOHT, which may be one mechanism through which triptolide has high activity in these tumors.

## Highlights

• SWI/SNF occupancy at super-enhancers occurs in the absence of SMARCA4 in SCCOHT
• SCCOHT super-enhancers are proximal to oncogenes and neuronal development genes
• SCCOHT patient-derived xenografts are highly sensitive to triptolide and minnelide
• Triptolide reduces the expression of many genes associated with super-enhancers

## Graphical abstract

**Figure:**
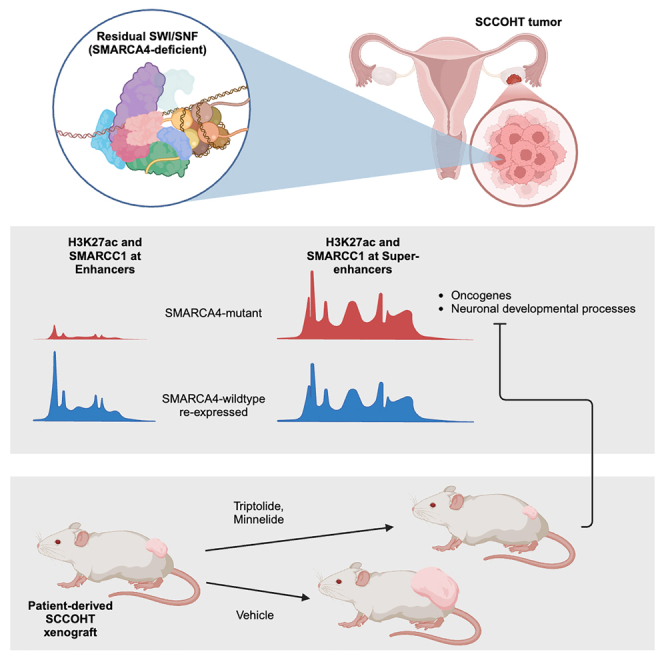

## Introduction

Small cell carcinoma of the ovary, hypercalcemic type (SCCOHT) is a rare and aggressive subtype of ovarian cancer that affects women at a mean of 24 years of age. Survival of SCCOHT remains poor, with a five-year overall survival rate estimated at 55%, 40%, 29%, and 0% for patients diagnosed at stages I, II, III, and IV, respectively.^1^ Two-thirds of patients are diagnosed at stage II–IV, necessitating better treatments to improve the survival of these patients with advanced disease. To date, treatment is based on multiple case reports with small patient cohorts. Standard treatment includes multi-agent chemotherapy and radiation, and treatment-refractory or relapsed patients have been treated with investigational agents.^2^

SCCOHTs are characterized by inactivating germline and/or somatic mutations in *SMARCA4* and concomitant protein loss in over 95% of tumors.^3^^,^^4^^,^^5^^,^^6^^,^^7^ SCCOHTs have low mutational burdens and lack other mutational genetic driver events.^3^^,^^5^^,^^6^^,^^8^ The loss of SMARCA4 (BRG1) and SMARCA2 (BRM) protein is pathognomonic for SCCOHT amongst other gynecologic cancers and histopathologic mimics.^4^ SMARCA4 and SMARCA2 are the two mutually exclusive ATPases of the switch/sucrose non-fermentable (SWI/SNF) chromatin remodeling complex whose enzymatic activities are necessary for energy-driven histone repositioning throughout the genome. Loss of SWI/SNF ATPases in SCCOHT has been shown to create dependencies on other epigenetic targets, such as polycomb repressive complex 2 (PRC2) and histone deacetylases (HDAC).^4^^,^^9^^,^^10^^,^^11^ The SMARCA4 subunit is particularly important for enhancer function.^12^^,^^13^ While the ATPase activity is essential for the regulation of almost a third of enhancers in SCCOHT cells, the residual SWI/SNF lacking SMARCA4 in SCCOHT is retained at 19% of enhancer sites.^14^ The function of residual SWI/SNF at these sites remains poorly characterized.

The unique activity of super-enhancers (SEs) has been implicated in SWI/SNF-mutant rhabdoid tumors, where SMARCB1-deficient SWI/SNF complex remains localized to SEs but not regular enhancers.^15^ SEs are large clusters of enhancers that can regulate the expression of many genes, driven through recruitment of RNA polymerase II, transcription factor complexes, such as transcription factor II H (TFIIH) and mediator, and histone modifications characteristic of open chromatin. While SEs in normal cells regulate genes important for cell lineage determination,^16^ SEs in many tumor types regulate oncogenes, leading to their constitutive overexpression.^17^ SCCOHT, ARID1A-deficient clear cell ovarian carcinoma, and SMARCA4/A2-deficient lung cancer cell lines are sensitive to bromodomain inhibitors that target proteins critical for the maintenance and function of SEs (*e.g.*, BRD4 in mediator complex).^18^^,^^19^^,^^20^^,^^21^ This suggests that SWI/SNF-deficient cancers may be dependent on SEs for their growth despite a lack of general enhancer function in SCCOHT. Characterizing SEs in SCCOHT could identify important therapeutic vulnerabilities in SCCOHT.

Here, we describe SE landscapes across SCCOHT cell line models, patient-derived xenografts (PDX), and tumors, including annotation of oncogenes associated with SEs. We further evaluated the efficacy of triptolide in SCCOHT models. Among triptolide targets is xeroderma pigmentosum type B (XPB),^22^ an ATP-dependent DNA helicase in the TFIIH transcription factor complex. TFIIH is essential for the assembly of transcriptional machinery and is found at high levels at SEs.^23^ Triptolide was effective in SCCOHT cell lines and strongly decreased the growth of SCCOHT PDX models. Ultimately, triptolide may be an effective therapeutic strategy for SCCOHT patients.

## Results

### SWI/SNF chromatin occupancy at enhancers vs. super-enhancers

**Figure 1:**
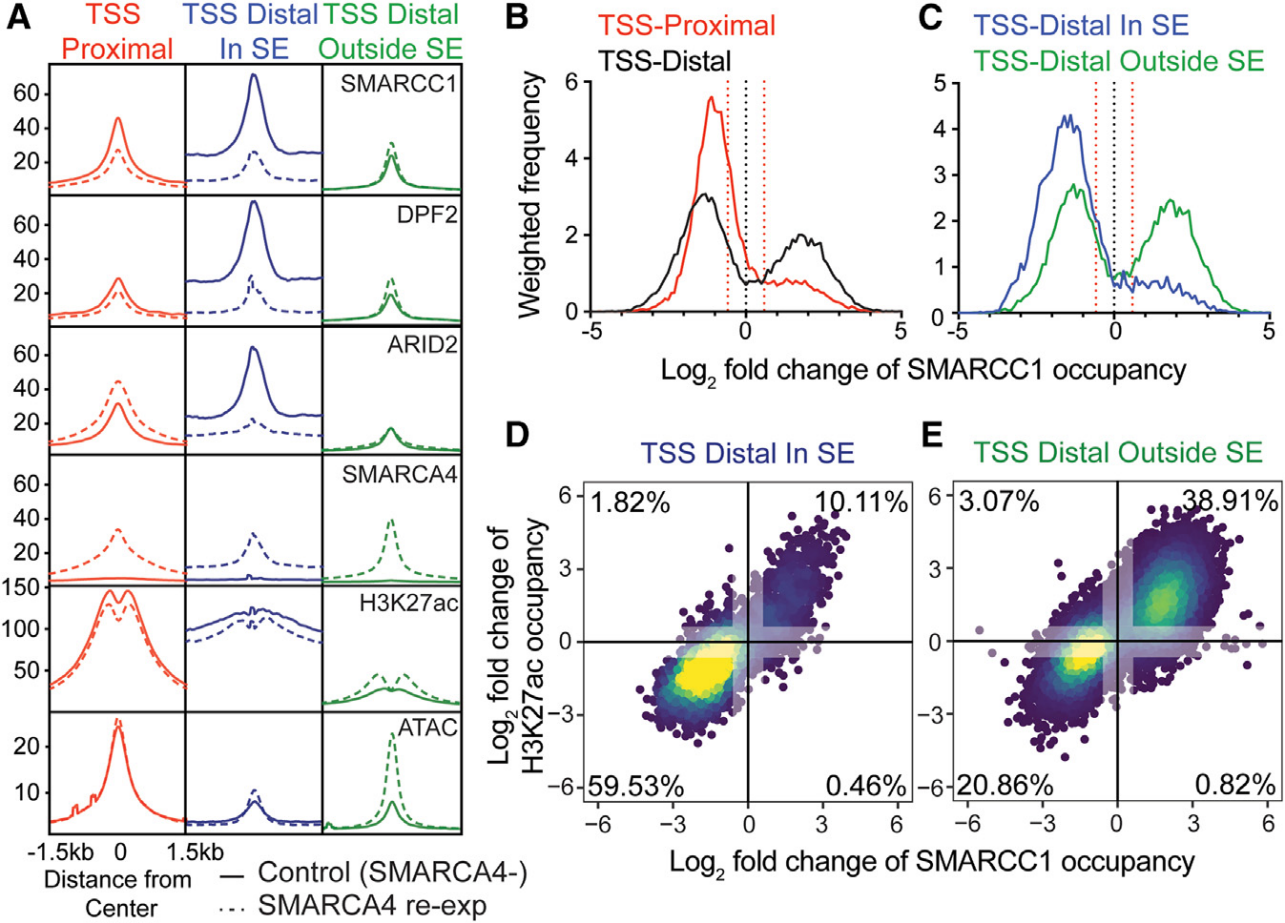
SWI/SNF binding at super-enhancer vs. non-super-enhancer sites SWI/SNF occupancy sites from BIN67 cells (ChIP data re-analyzed from Pan et al.^14^ are annotated as proximal (overlapping TSS, 17,866 sites) or distal (>2 kb; 18,997 sites) to TSS. Distal sites are separated by those overlapping SE sites (In SE; 4,518 sites), as determined by ROSE on H3K27ac ChIP data, and those non-overlapping those sites (Outside SE; 14,479 sites). (A) Average chromatin occupancy profile of SWI/SNF subunits SMARCC1, DPF2, ARID2, and SMARCA4, as well as H3K27ac and ATAC signal at SWI/SNF-occupied sites, with and without SMARCA4 re-expression. Average ChIP/ATAC signal across region type spanning 1.5kb up- and downstream of the peak center is shown. (B and C) Histograms of log_2_ fold-change of SMARCC1 IP signal in SMARCA4 re-expression vs. control expression BIN67 cells at (B) proximal vs. distal SWI/SNF binding sites or (C) distal sites within or outside SE, as described in (A). Histogram frequencies are weighted based on the total number of sites in each group (*e.g.*, number of proximal vs. distal SWI/SNF binding sites). Positive values indicate more SMARCC1 occupancy at sites defined as SWI/SNF occupied sites from either control or SMARCA4-re-expression conditions, and negative values indicate less SMARCC1 occupancy at those sites with SMARCA4 re-expression. +/−1.5-fold change is demarcated by the dotted red line. (D and E) Log_2_ fold-change of SMARCC1 occupancy vs. H3K27ac at each SWI/SNF site. Plot is colored based on the density of points, with yellow being the highest density and blue being the lowest. The percent of total SWI/SNF sites that had +/− 1.5-fold change in SMARCC1 and H3K27ac is annotated, and values between −1.5 and 1.5 for each are grayed out to indicate which values are considered in this calculation. See also Figure S1.

The residual SWI/SNF complex in SMARCA4-deficient SCCOHTs has been shown to have dysregulated chromatin occupancy in a manner that largely depends on ATPase loss.^14^ This previous study showed differences in SWI/SNF occupancy and subunit composition between transcription start site (TSS)-proximal and -distal sites. ARID2-containing Polybromo-associated BAF (PBAF) complexes tend to localize to TSS-proximal and DPF2-containing BRG-/BRM-Associated Factor (BAF) complexes tend to localize to TSS-distal sites upon SMARCA4 restoration.^14^ In SMARCB1-deficient rhabdoid tumors, loss of SMARCB1 caused differential effects on residual SWI/SNF occupancy at distal enhancers vs. SEs.^15^ We sought to determine whether disruption of the SWI/SNF ATPase subunit in SCCOHT similarly causes differential effects at these distinct distal sites by re-examining the data produced by the Kadoch group in an SCCOHT cell line, BIN67 (Figures 1A and S1). This analysis largely recapitulated the distinct BAF vs. PBAF complex localization at proximal and distal sites upon SMARCA4 re-expression. However, we found that distal sites within SE regions displayed an altogether different composition of SWI/SNF, where residual SWI/SNF occupancy was, on average, higher, including both DPF2 and ARID2 signal. Interestingly, SMARCA4 re-expression led to a decrease in overall SWI/SNF occupancy at SE sites, as opposed to increases in PBAF at TSS-proximal sites and BAF at TSS-distal sites outside SEs. Histone 3 Lysine 27 acetylation (H3K27ac) signal was also reduced at sites of SWI/SNF occupancy in SEs upon SMARCA4 re-expression. Interestingly, chromatin accessibility, as measured by Assay for Transposase-Accessible Chromatin and sequencing (ATAC-seq), was slightly increased at these SE sites. However, looking at the distribution of SWI/SNF occupancy at SEs, the regions with decreased H3K27ac occupancy appeared to have modest changes in accessibility compared to SWI/SNF occupancy sites with increases in H3K27ac, which displayed great increases in accessibility following SMARCA4 re-expression (Figure S1). In fact, three times more SWI/SNF occupancy sites within SEs had decreases in H3K27ac of > 2-fold than SWI/SNF occupancy sites in proximal or distal non-SE sites.

Interestingly, SWI/SNF sites generally showed changes in the core subunit SMARCC1 occupancy with SMARCA4 re-expression (Figures 1B and 1C). Distal sites more of an increase in SMARCC1 occupancy after SMARCA4 re-expression than proximal sites (Figure 1B). However, distal sites within SEs displayed even stronger decreases in SMARCC1 occupancy, with only a minority of sites displaying increased SMARCC1 occupancy (Figure 1C). In addition, upon SMARCA4 re-expression, SMARCC1 occupancy changes correlated with H3K27ac occupancy changes across distal SWI/SNF sites (Figures 1D and 1E). Distal sites in SEs, however, were more likely to have concomitant decreases in SMARCC1 and H3K27ac across sites, with 59.53% showing dual decreases in SE sites vs. only 20.86% of non-SE distal sites. Together, these data suggest that the SMARCA4 deficiency creates a unique SE state that may be important for the epigenetic reprogramming in SCCOHT.

### Landscape of super-enhancers in SCCOHT cell lines

**Figure 2:**
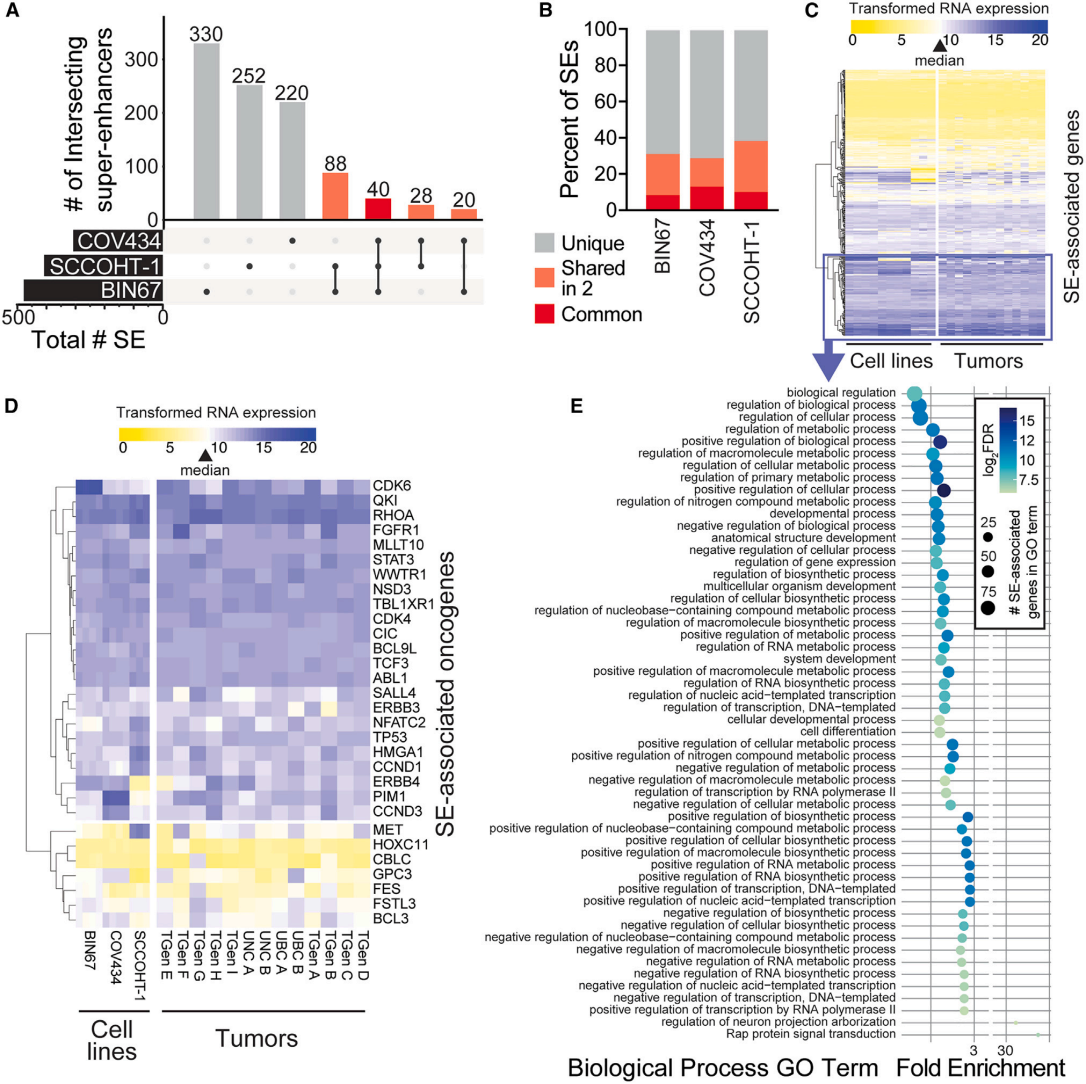
SE landscape and associated gene expression in SCCOHT cell lines and tumors (A) UpSet plot of overlapping SE regions in three SCCOHT cell lines. SEs were identified by ROSE or CREAM across three replicate H3K27ac CUT&RUN experiments in each cell line and merged for each cell line, then compared using Intervene. Overlapping SE regions are shown in shades of red, with bold red representing overlap in all three cell lines. (B) For each cell line, the percentage of SEs classifying as “unique,” “shared in 2”, or “common” are shown. Colors match those in (A). (C and D) Unsupervised hierarchical clustering of variance stabilizing transformed-TPM RNA levels from RNA-seq of (C) 558 genes within 50kb of the 176 SEs identified in more than one SCCOHT cell line or (D) 30 oncogenes within 50kb of any SE identified in SCCOHT cell lines. Three to four replicates of RNA-seq in SCCOHT cell lines (left) or single RNA-seq analyses from thirteen SCCOHT tumors (right) are shown. Sample order is the same in each panel. The color scale is centered on a white value indicating the median expression value of all protein-coding genes across samples (indicated by a triangle in legend), such that purple indicates higher expression and yellow indicates lower expression than any other gene annotated in the genome. The top two clusters in (C) with high expression across cell lines and tumors are highlighted with the purple box and were selected for further gene ontology analysis in (E). (E) Gene ontology (GO) analysis of highly expressed, SE-associated genes using Panther. Bubble plot shows the log_2_ enrichment score on the x axis, the log_2_ false discovery rate (FDR) as bubble color, and the size of the SE-associated genes within each term as the bubble size. See also Table S1.

To more comprehensively examine SE landscapes in SCCOHT, we performed H3K27ac Cleavage Under Targets and Release Using Nuclease (CUT&RUN) in the three available SCCOHT cell lines, BIN67, COV434, and SCCOHT-1, all of which carry biallelic inactivating mutations in SMARCA4.^5^^,^^37^ Combining results from two SE callers, ROSE and CREAM, we identified 478 SEs in BIN67, 308 SEs in COV434, and 408 SEs in SCCOHT-1 (Figure 2A). While the majority of SEs (61.8–71.4%) were unique to each cell line, a total of 176 (28.6–38.2%) of SE regions overlapped in more than one cell line, with 40 regions (8.4–13.0%) overlapping in all three SCCOHT cell lines (Figure 2B). We examined gene expression from SCCOHT cell lines and tumor RNA-seq of the 401 genes located within 50 kb of the 176 SE regions detected in at least 2 SCCOHT cell lines (Figure 2C).

We identified 30 oncogenes within proximity to SEs identified in any of the SCCOHT cell lines. 85% of these oncogenes were expressed higher than the protein-coding gene median (Figure 2D). A notable cluster of oncogenes was highly expressed across all three cell lines and 13 SCCOHT tumors. These included *CDK4*, *CDK6*, and *FGFR1*, which were found previously to be therapeutic targets in SCCOHT models.^24^^,^^25^ *ERBB3* and *ERBB4* were also high and associated with SEs.

Gene ontology (GO) analyses identified the enrichment of developmental and metabolic biological processes among the most highly expressed clusters of SE-associated genes (purple box in Figure 2C) in SCCOHT (Figure 2E; Table S1). The most statistically significant GO term was “developmental process,” which was also related to significant terms “anatomical structure development,” “nervous system development,” “multicellular organism development,” “system development,” and “cellular developmental process.” This is interesting, given the roles of SEs in normal cell processes, which include lineage specification. Other themes in GO terms included general biologic and metabolic process regulation, as well as more specific processes, including “IRE1-mediated unfolded protein response”.

### SALL4 and MLLT10 expression is high in SCCOHT

**Figure 3:**
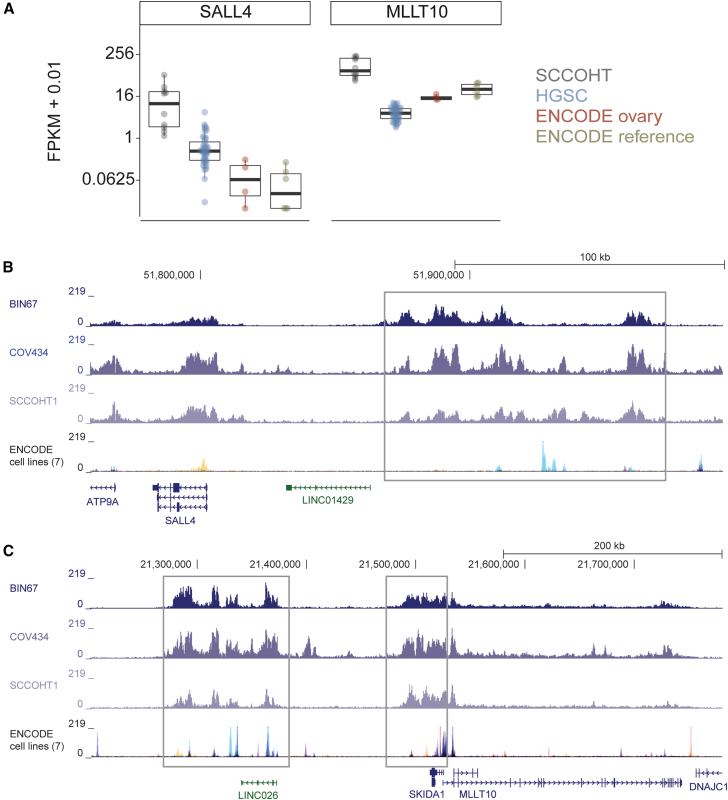
Oncogenes *SALL4* and *MLLT10* are highly expressed in SCCOHT through a unique super-enhancer (A) RNA expression of *SALL4* and *MLLT10* from RNA-seq on SCCOHT tumors, age-matched TCGA high-grade serous ovarian carcinoma (HGSC) tumors, normal ovary tissue from ENCODE (ENCODE ovary), and ENCODE reference cell lines (ENCODE reference). FPKM + 0.01 is plotted on a logarithmic scale, with individual circles representing each sample and the box and whiskers plot summarizing the median value (heavy line), upper and lower 25% quartiles (box limits), and error bars showing the highest and lowest values. (B and C) H3K27ac tracks at *SALL4* (B) and *MLLT10* (C) SEs. The gray boxes indicate the common SE regions between the 3 SCCOHT cell lines. The H3K27ac ChIP-seq tracks from the ENCODE reference cell lines (GM12878, H1-hESC, HSMM, HUVEC, K562, NHEK, and NHLF) are shown on the bottom track for comparison, with each track overlaid according to UCSC Genome Browser’s default settings and colors. See also Figures S2–S4.

We examined the 30 oncogenes associated with SEs between the 3 SCCOHT cell lines for expression in SCCOHT relative to age-matched The Cancer Genome Atlas (TCGA) high-grade serous ovarian carcinoma (HGSC) tumors, normal ovary tissue from the Encyclopedia Of DNA Elements (ENCODE;ENCODE ovary), and ENCODE reference cell lines (ENCODE reference). Patterns of specificity of high expression differed among these 30 genes (Figure S2). *SALL4* and *MLLT10* were the most selectively expressed in SCCOHT tumors relative to either HGSC or normal samples (Figure 3A). Consistent with this observation, the SE regions upstream of *SALL4* and *MLLT10* had consistent high H3K27ac signal in SCCOHT cell lines, but reduced H3K27ac signal across 7 ENCODE reference cell lines (Figures 3B and 3C). An expanded H3K27ac signal was observed in comparison to an expanded set of normal and malignant samples (Figure S3), including putative cells-of-origin of SCCOHT tumors (neuronal precursors), epithelial ovarian cancers (fallopian tube epithelium), other gynecologic normal tissues, and cancer cell lines. We also examined SALL4 expression in SCCOHT tumors by immunohistochemistry (IHC). In an SCCOHT tissue microarray (TMA) with 14 SCCOHT patients, 8 (57%) had positive SALL4 staining (Figure S4). Staining was typically weak and in <20% of the cells in a given area. SALL4 staining was focally strong in one tumor sample.

### Triptolide affects gene expression and super-enhancers in SCCOHT

**Figure 4:**
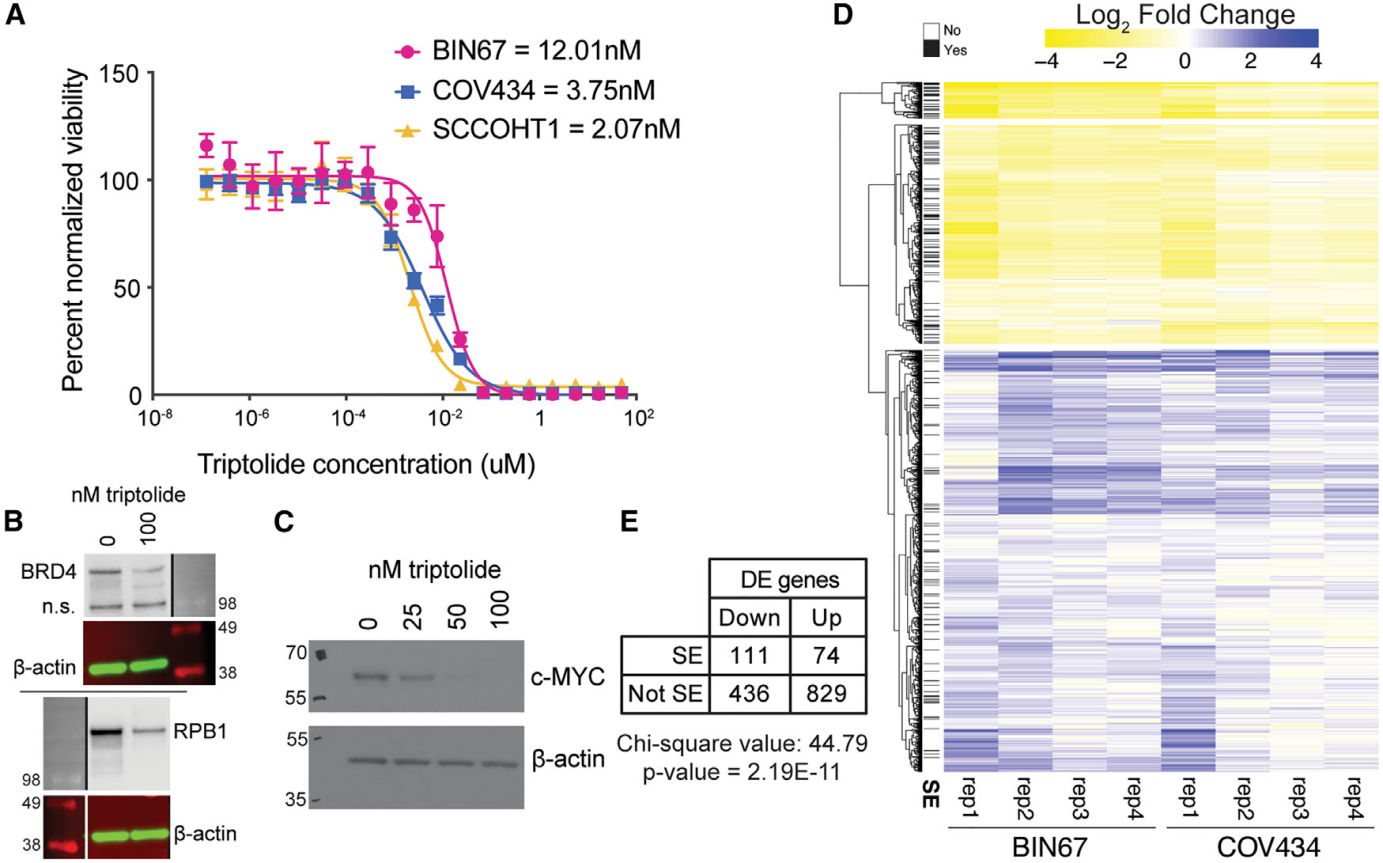
Triptolide inhibits super-enhancers in SCCOHT (A) Drug dose-response assay on SCCOHT cell lines treated with triptolide for 72 h. IC_50_s for each SCCOHT cell line are provided in the key. Data are represented as mean ± SEM. (B) BRD4 and RPB1 western blots on BIN67 cells treated with vehicle (0.01% DMSO) or 100 nM Triptolide for 24 h β-actin serves as a loading control. Molecular weight markers on the same blot are shown, and sizes in kDa are labeled (imaged in different channels). N.s. = non-specific band. Black/white lines indicate that molecular weight markers were imaged in a separate channel and/or cropped lanes. (C) c-Myc western blot on BIN67 cells treated with serial dilutions of triptolide for 24 h β-actin serves as a loading control. Molecular weight markers on the same blot are shown, and sizes in kDa are labeled (imaged in different channels). (D) Differential gene expression in SCCOHT cell lines BIN67 and SCCOHT1 after 6 h of treatment with triptolide. Yellow represents a decrease in expression with triptolide treatment, and purple represents an increase. Genes within 50 kb of an SE are denoted on the left with a black line (SE column). (E) Summary of differentially expressed genes from data in (D). DE genes were annotated by the direction of gene expression change and whether they were 50 kb from an SE. A chi-square test was run to determine whether there was a statistically significant association between direction of change and being near an SE. See also Table S2.

We next assessed whether triptolide, a drug shown to target the XPB subunit of the TFIIH complex,^22^ might alter super-enhancers and their associated genes in SCCOHT. Using drug dose-response assays, triptolide potently inhibited the growth of all three SCCOHT cell lines, BIN67, SCCOHT-1, and COV434, with half-maximal inhibitory concentrations (IC_5__0_) in the low nanomolar range (2–12 nM) (Figure 4A). Triptolide treatment reduced global protein levels of BRD4 and RPB1 (Figure 4B), which are important for SE function through their participation in the mediator and RNA polymerase II complexes, respectively. These global changes have been observed in other models in which triptolide targets XPB.^22^ As one of the most commonly SE-associated oncogenes, c-Myc protein levels decreased in a dose-dependent manner with triptolide treatment (Figure 4C).

To determine whether triptolide treatment is associated with changes in SEs in SCCOHT cells following treatment, we performed H3K27ac CUT&RUN and RNA-seq in BIN67 and COV434 cells after a 6-h treatment with triptolide. A total of 974 and 460 up-regulated genes and 407 and 155 down-regulated genes were significantly changed following triptolide treatment in BIN67 and COV434 cell lines, respectively, for a total of 903 and 547 up- and down-regulated genes overall (Figures 4D and 4E; Figures S5A and S5B; Table S2). The ratio of SE-associated genes that were down-regulated vs. upregulated (1.50) was much higher than for non-SE genes (0.52), which was statistically significant (Figure 4E).

Interestingly, H3K27ac was largely static at the same 6-h time point after triptolide treatment, suggesting that the effects of triptolide treatment occurred downstream of the establishment of the open chromatin environment (Figure S5). Only one peak was significant, increasing only in BIN67, and it was not associated with an SE. By 16 h, H3K27ac starts to decline at some SEs (Figure S5). COV434 showed fewer downregulated SE peaks than observed in BIN67, likely due to the overall fewer SEs near oncogenes identified in COV434. In BIN67, H3K27ac peaks within SEs that displayed decreases included those near *SALL4*, *TMSB4X*, *MLLT10*, *PIM1*, *BCL3*, *CBLC*, *HOXC11*, and *MALAT1*.

### SALL4, PIM1, and MLLT10 expression is significantly reduced by triptolide

**Figure 5:**
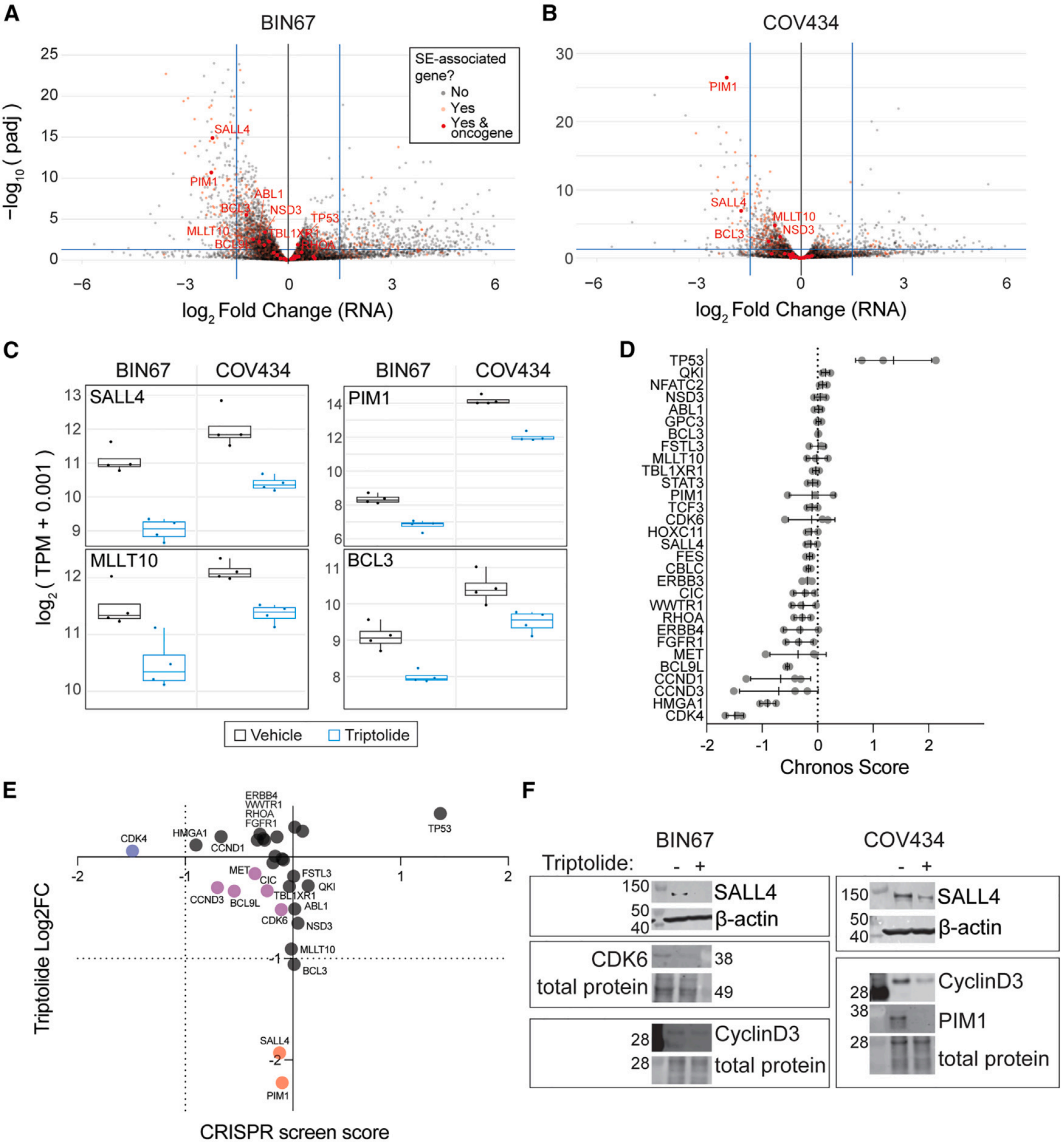
Triptolide inhibits the expression of SE-associated oncogenes (A and B) Volcano plots of differential gene expression of BIN67 (A) and COV434 (B) after treatment with triptolide for 6 h. Blue bars denote *p* value = 0.05 and log_2_ Fold Change = ±1.5. SE-associated genes are denoted by red dots, with non-transparent dots indicating SE-associated oncogenes. Those meeting either cutoff are labeled with gene names. (C) RNA expression of notable SE-associated oncogenes with repression of expression by triptolide. Box and whiskers plot summarizes the median value and upper and lower 25% quartiles (box limits), and error bars show the highest and lowest values. (D) Mean CRISPR screen cell viability score (Chronos score) for SE-associated oncogenes in BIN67, COV434, and SCCOHT1. Error bars represent +/− one standard deviation. (E) SCCOHT cell line average DepMap CRISPR screen score versus average triptolide treated log2 fold change in RNA expression of SE-associated oncogenes. Gene names are as indicated, except for clusters with no appreciable difference in either assay. Genes highlighted in blue indicate CRISPR screen-specific hits, red indicates differential expression hits, and purple indicates some intermediate hits on both screens. (F) Western blots for SE-associated oncogenes in BIN67 and COV434 cells treated with 50 nM triptolide for 24 h. For SALL4, beta-actin serves as a loading control, and for all other proteins, total protein stain was used as a loading control. Molecular weight markers on the same blot are shown, and sizes in kDa are labeled. See also Figure S5.

We examined the expression of SE-associated oncogenes to address whether triptolide also reduced their abundance at the transcriptional level. Overall, the expression of SE-associated oncogenes either decreased or had minimal change (Figures 5A and 5B). In both BIN67 and COV434, *PIM1* and *SALL4* were the only SE-associated oncogenes with statistically significant decreased expression (Figures 5A–5C). Many of the SE-associated oncogenes displayed a trend toward decreased expression, although most did not pass the threshold of statistical significance (Figure 5C).

To determine whether SE-associated oncogenes are essential for the growth of SCCOHT tumors, we looked into publicly available genome-wide CRISPR knockout (KO) data on the three SCCOHT cell lines. DepMap’s Achilles Project has included the three SCCOHT cell lines in their CRISPR screen data. Based on the average Chronos score for the SE-associated oncogenes, they were more likely to have a detrimental effect on cell growth (Figure 5D), though the effect size of targeting most single SE-associated genes was not large. Interestingly, *TP53* knockout increased the growth of SCCOHT cell lines, consistent with its canonical tumor suppressor activity in cancer. We left *TP53* in the analysis for two reasons: (1) to remain unbiased, as the Catalogue of Somatic Mutations In Cancer (COSMIC) identified it as a potential oncogene in some cancer contexts, and (2) based on specific observations in SMARCB1-deficient rhabdoid tumors which show that TP53 is a synthetic lethal target.^26^^,^^27^ The correlation between RNA expression changes following triptolide treatment and the effect of knockout in SCCOHT cell lines was not linearly associated, with an R-squared value of 0.0022 (Figure 5E). However, a few genes did have concordant negative effects on both gene expression and growth, including *CCND3*, *BCL9L*, *MET*, *CIC*, and *CDK6*.

To confirm the downregulation of the SE-associated oncogenes, protein abundance was examined by western blotting. In BIN67 cells, we confirmed that triptolide reduced SALL4, CDK6, and CyclinD3 protein levels. In COV434, we confirmed the downregulation of SALL4, CyclinD3, and PIM1 (Figure 5F). PIM1 was not detected in BIN67, and CDK6 was not detected in COV434.

### Triptolide and minnelide are potent in preclinical models of SCCOHT

**Figure 6:**
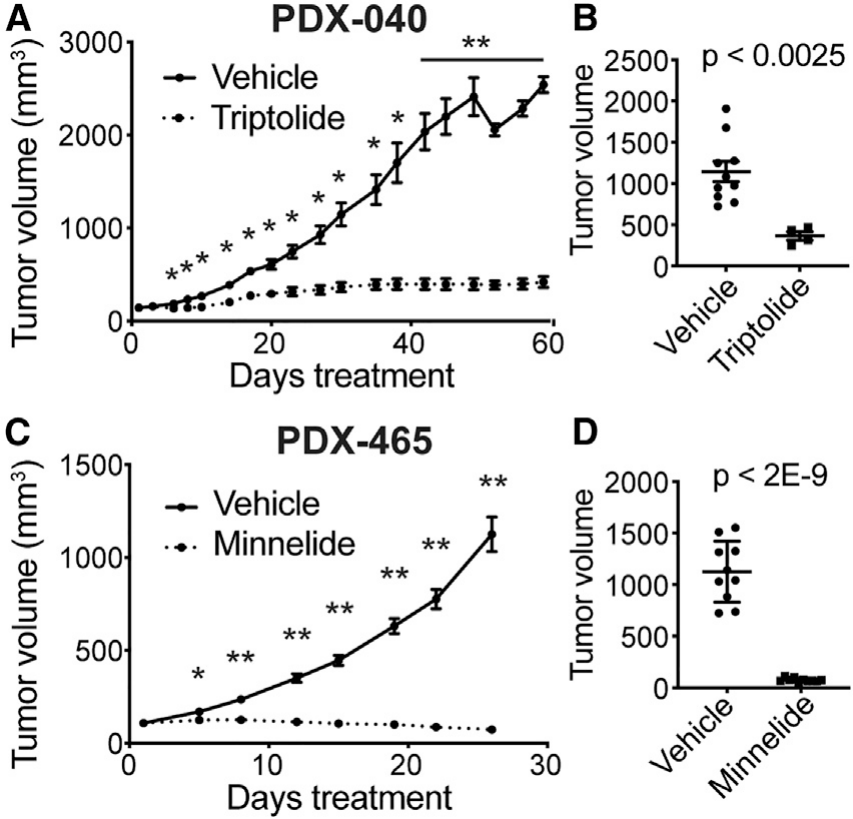
Efficacy of triptolide/minnelide in SCCOHT *in vivo* (A and B) SCCOHT PDX model 040 treated with vehicle (DMSO i.p.; *N* = 10) or triptolide (0.6 mg/kg i.p., QD days 1–5, QOD days 10–60, *N* = 4). (A) Tumor volume measurements from initiation of treatment (day 1) to study end (day 60). Multiple t-tests were used to determine the *p* value. ∗ <0.05, ∗∗ <0.0001. (B) Tumor volume measurements at day 30. Student’s t test was used to determine the *p*-value. (C and D) SCCOHT PDX model 465 treated with vehicle (PBS i.p., *N* = 10) or minnelide (0.42 mg/kg i.p., QD, *N* = 10). (C) Tumor volume measurements from initiation of treatment (day 1) to study end (day 26). A paired t test was used to determine the *p* value. (D) Tumor volume measurements at day 26. Student’s t test was used to determine the *p* value. Data are represented as mean ± SEM throughout. See also Figure S6.

To determine the preclinical efficacy of triptolide for SCCOHT, we performed *in vivo* efficacy studies in PDX models PDX-040 and PDX-465.^25^ Triptolide treatment of PDX-040 reduced tumor growth by 77.9% at 30 days of treatment and continued to repress tumor growth through 60 days of treatment (Figures 6A and 6B; individual mouse tumor weights in Figures S6A and S6B). Since triptolide is known to be less bioavailable due to poor water solubility, we also tested the activity of its more water-soluble prodrug, minnelide.^28^ Minnelide reduced the growth of PDX-465 tumors, with an overall reduction of 31.5% in tumor volume since the initiation of treatment, whereas the vehicle-treated PDX-465 tumors had increased in volume by 1,030% (Figures 6C and 6D). Overall, mice tolerated both triptolide and minnelide, with mice maintaining stable body weight over the course of treatment (Figures S6C and S6D). Starting tumor volumes were also equivalent in control and treatment groups, demonstrating that mice were appropriately randomized at the initiation of treatment (Figures S6E and S6F).

## Discussion

This report is the first to characterize the SE landscape of SCCOHT, an ovarian cancer subtype driven by widespread epigenetic dysregulation due to the loss of the SWI/SNF chromatin remodeling complex ATPase subunits SMARCA4 and SMARCA2. We demonstrate that the 30 oncogenes found near SEs detected in more than one SCCOHT cell line are also highly expressed in an independent cohort of SCCOHT tumors. Expression of these oncogenes, such as *SALL4* and *MLLT10*, distinguish SCCOHT tumors from HGSC tumors, normal ovary, and other reference cell lines, suggesting their importance broadly in the oncogenic processes of SCCOHT.

SALL4 was associated with an SE in both BIN67 and SCCOHT-1 cells. Given its role as a transcription factor in early embryonic development and maintaining cell stemness, SALL4 may be crucial for maintaining SCCOHT’s undifferentiated phenotype. The low abundance of SALL4 expression in differentiated adult tissues, along with its potential role as an immunogenic antigen,^29^ could at least partially underlie the perplexing sensitivity of low-mutation burden SCCOHTs to immune checkpoint inhibitors.^30^ This theory is yet untested in SCCOHT tumors but is worth exploring in future directions. One immunohistochemistry study has also validated the high expression of SALL4 in SCCOHT compared to high-grade serous ovarian cancer.^31^ Rhabdoid tumors have also been shown to have an SE resident at an overlapping position upstream of the SALL4 gene and similarly depend upon its expression for rhabdoid tumor cell line growth.^15^

In our reanalysis of SCCOHT data with simultaneously prepared SWI/SNF subunit and H3K27ac chromatin immunoprecipitation and sequencing (ChIP-seq) and ATAC-seq, we observed that SWI/SNF occupancy sites in SEs differed from those of enhancer sites. Following SMARCA4 re-expression, SMARCA4 occupancy increased at SE sites, while other subunits showed overall reductions. This pattern is distinct from those observed at transcription start sites or other distal binding sites. Additionally, the total H3K27ac signal at the SE sites was reduced. Together, this supports a model where SMARCA4 loss in SCCOHT strengthens SE sites, where SMARCA4-deficient SWI/SNF complexes reside despite their lack of ATPase function. Wang et al. similarly looked at SWI/SNF complex occupancy at SEs versus other distal sites in SMARCB1-deficient rhabdoid tumors.^15^ In rhabdoid tumors, SMARCB1 re-expression increased SWI/SNF occupancy at distal sites generally but not at SE sites, where it remained unchanged. Our data demonstrate a loss of SWI/SNF occupancy at SEs with SMARCA4 re-expression, which is in contrast to what is observed in rhabdoid tumors. In rhabdoid tumors, the loss of SMARCB1 does not interfere with the non-canonical BAF (ncBAF) complex, which requires a functional ATPase subunit. However, in SCCOHT tumors, the SWI/SNF complex only exists in a residual form distinct and less abundant than ncBAF.^32^ Further, the rhabdoid tumor data did not show a corresponding loss of H3K27ac signal at SEs. Thus, it is highly likely that the underlying differences observed at SEs between SCCOHTs and rhabdoid tumors are driven by the different forms of the SWI/SNF complex predominant based on the specific subunit affected.

The exceptional growth inhibition by triptolide in SCCOHT models exceeds responses observed by other published inhibitors by us and others.^9^^,^^10^^,^^11^^,^^18^^,^^24^^,^^25^ Among the known targets of triptolide is XPB, a subunit of the TFIIH complex. Inhibition of XPB activity may account for the observed decreases in the global expression of its associated transcriptional machinery, including BRD4 and RNAPII subunit RPB1, as well as expression of several oncogenes near SEs identified in SCCOHT cell lines. Noel et al. observed similar effects of triptolide on H3K27ac at SEs in pancreatic cancer.^33^ In their co-culture model, they observed more dramatic transcriptional effects on the stromal microenvironment than the tumor cells themselves, yet dramatic effects on cancer growth. While our data are consistent with triptolide functioning through XPB/TFIIH, we cannot exclude the possibility that it also functions through other previously identified targets. Nuclear factor kappa B (NF-kB) is one alternative target. We have previously examined NF-kB localization in SCCOHT cell lines using nuclear vs. cytoplasmic fractions, as NF-kB is translocated to the nucleus upon activation. NF-kB was entirely cytoplasmic in all three SCCOHT cell lines (data not shown), which would suggest that its activity is very low in SCCOHT. In addition, XPB/TFIIH is also critical for DNA damage repair processes through nucleotide excision repair. We cannot exclude this as a possibility but consider this pathway relatively low for SCCOHT tumors in the absence of DNA damaging agent co-treatment in our model systems, as SCCOHT tumors have exceptionally low tumor mutation burden^8^ and thus are expected to have low levels of endogenous DNA damage compared to most other tumor types.

In our data, we surprisingly see that genes strongly downregulated by short triptolide treatment were not the same genes that reduced growth in genome-wide CRISPR KO screens performed by DepMap in the same cell lines. However, these findings may indicate a multi-gene effect, where the loss of SMARCA4 in SCCOHT leads to more SE activity at oncogenes at several sites. This is supported by our analysis of the aforementioned data, where re-expression of wild-type SMARCA4 resulted in a strongly reduced H3K27ac signal at around two thousand SWI/SNF occupancy sites. It is possible that gene-by-gene screens do not have strong effects on their own; however, simultaneously targeting SEs genome-wide using triptolide has stronger effects. Additionally, CRISPR KO is related specifically to cell viability in a knock-out setting performed at late time points, whereas triptolide gene downregulation was observed at only 6 h after treatment to avoid off-target effects. This will lead to an over-representation of CRISPR KO hits that are due to downstream effectors of triptolide and/or SE targets, as well as down-regulated genes in response to triptolide that have no significant effect on growth. Thus, the union of these two screens is likely to reveal triptolide targets most relevant for viability in SCCOHT.

Recently, Shorstova et al. described the sensitivity of SMARCA4/A2 dual-deficient cell lines, including the SCCOHT cell line SCCOHT-1, to the bromodomain inhibitors JQ1 and OTX015. Interestingly, they found that *ERBB3* is repressed following treatment with OTX015. We found *ERBB3* to be in close proximity to SEs in SCCOHT-1 cells, providing a potential mechanism for the effect of bromodomain inhibitors. We also found both *CDK4* and *CDK6* near SEs in SCCOHT cell lines. We also previously demonstrated the sensitivity of SCCOHTs to receptor tyrosine kinase inhibitor ponatinib.^25^ *FGFR1*, one target of ponatinib, is also associated with an SE in SCCOHT cell lines.

The efficacy of SE inhibitors in cancer has been debated based on the potential side effects of targeting general transcription machinery in cancer cells and in normal tissues, which has been reviewed elsewhere for triptolide specifically.^34^ The possibility exists that clinically relevant doses of these inhibitors have more specific effects on SEs, as these non-saturating doses likely primarily affect regions of high transcriptional activity. Known toxicities of triptolide include male infertility and damage to the liver, kidneys, and cardiovascular system,^34^ with effects on the liver being the most prominent. We did not observe general toxicity in our PDX efficacy models after treatment with doses of triptolide and minnelide that resulted in stable disease or tumor regression. Mice continued to gain body weight even while on treatment for up to two months.

Minnelide is being tested in clinical trials for human cancers, with two completed but not yet reported trials in pancreatic (NCT03117920; phase II) and gastrointestinal tumors (NCT01927965; phase I). Additional trials are also currently recruiting: three phase I, two phase Ib, and a phase II trial in pancreatic cancer. Full reports of these studies have not yet been released. Early phase I results demonstrate reasonable tolerability of the drug, with some cases of hematologic toxicity. Early reports from a pharmacokinetic study of minnelide report two pancreatic cancer patients with some clinical benefit for seven months.^35^ Based on our data that demonstrates a unique feature of SE dysregulation following SMARCA4 loss, associated gene expression programs regulating oncogenes, and exceptional responses observed in animal models, triptolide should be considered a promising treatment for SCCOHT patients.

### Limitations of the study

While our data supports triptolide targeting super-enhancers as the mechanism of action in SCCOHT, we did not definitively link XPB/TFIIH inhibition at super-enhancers. Two questions remain to mechanistically link its action, which are outside the scope of this study. First, since TFIIH is generally important for transcription, uncoupling effects of TFIIH at super-enhancers from those at promoters remains challenging to experimentally determine. Nevertheless, super-enhancers mark genes with high gene expression that are poised to be targeted by triptolide at non-saturating doses and thus may still serve as a biomarker of sensitivity. Second, other triptolide targets, while unlikely in the SCCOHT system due to previously mentioned features of this cancer, cannot be excluded based on our data and may even function in a multifactorial manner. Experiments to specifically test the contributions of SE-associated genes to the sensitivity to triptolide were not performed here due to the limited translational yield anticipated from such studies but will be important to perform to determine the specific mechanism of triptolide.

We opted to use subcutaneous patient-derived xenograft models for drug efficacy studies, as they are generally the field standard and allow for simple monitoring of tumor growth. A more robust approach would be to use a transgenic model or orthotopic xenograft. Transgenic models of SCCOHT do not yet exist. In addition, orthotopic models would require the manipulation of SCCOHT PDX models to express a luciferase or other reporter for tumor growth monitoring over time. Because we did not anticipate the drug effect to be significantly impacted by the tumor microenvironment according to our model, we opted for subcutaneous implantation as a reasonable approximation. We were also unable to assess toxicity in other organ systems in our model specifically. For the doses used in our animal model, it would be important to determine whether heart, liver, or kidney toxicity exists, but this is the subject of future directions.

## Resource availability

### Lead contact

Requests for further information and resources should be directed to and will be fulfilled by the lead contact, Jessica D. Lang (jessica.lang@wisc.edu).

### Materials availability

This study did not generate new unique reagents.

### Data and code availability

• H3K27ac CUT&Tag FASTQ, BIGWIG, and NARROWPEAK files for untreated BIN67, SCCOHT1, and COV434 have been deposited at GEO: GSE232727 and are publicly available as of the date of publication. H3K27ac CUT&Tag FASTQ, BIGWIG, and NARROWPEAK files for triptolide or vehicle-treated cells have been deposited at GEO: GSE232745 and are publicly available as of the date of publication. RNAseq FASTQs and TPM count matrix on triptolide or vehicle-treated cells have been deposited at GEO: GSE232831 and are publicly available as of the date of publication. RNAseq TPM count matrix on six SCCOHT tumors have been deposited at GEO: GSE216801 and are publicly available as of the date of publication. FASTQ files for the six tumor RNA-seq have been deposited at dbGaP: phs001528.v2.p1 and are available through controlled access. This paper analyzes existing, publicly available data, accessible at dbGaP: phs001528.v1.p1, GEO: GSE109919, and GEO: GSE117734.
• This paper does not report original code.
• Any additional information required to reanalyze the data reported in this paper is available from the lead contact upon request.

## Acknowledgments

This work is supported by the 10.13039/100000002National Institutes of Health (R01CA195670 and R01CA195670-S2 to D.G.H., J.T., and B.W. and K99CA234391 to J.D.L.), the 10.13039/501100000024Canadian Institutes of Health Research (CIHR PJT-462168 to Y.W.), the Terry Fox Research Institute Initiative New Frontiers Program in Cancer (D.G.H.), the Marsha Rivkin Center for Ovarian Cancer Research, the Ovarian Cancer Alliance of Arizona, the Small Cell Ovarian Cancer Foundation, Colleen’s Dream Foundation, and philanthropic support to the TGen Foundation. Thank you to Karthigayini Sivaprakasam for her bioinformatics advice. We would also like to thank the SCCOHT patients, their families and communities, and the clinicians who have contributed significantly to the motivation and feasibility of this work.

## Author contributions

Conceptualization, J.D.L., W.P.D.H., D.G.H., B.E.W., and J.M.T.; methodology, W.S.; software, S.S.; investigation, J.D.L., W.S., S.S., N.A.H., R.K., A.N.K., J.R.W., S.J.F., K.A.O., E.A.R., and Y.W.; resources, A.N.K., F.K.F.K., F.K., and V.L.Z.; formal analysis, J.D.L., S.S., and A.N.K.; writing – original draft, J.D.L.; writing – review and editing, J.D.L., A.N.K., P.P., E.A.R., Y.W., and B.E.W.; supervision, J.D.L., W.P.D.H., and J.M.T.; project administration, J.D.L., W.P.D.H., and J.M.T.; funding acquisition, J.D.L., D.G.H., B.E.W., and J.M.T.

## Declaration of interests

Since their contributions to this manuscript, several authors have new institutional affiliations not listed in the author affiliations: William Selleck - current affiliation: CellProtein Sciences, LLC (consultant); Shawn Striker - current affiliation: The Ohio State University; Nicolle Hipschman - current affliation: University of Arizona; Salvatore J Facista - current affiliation: Anivive; William P. D. Hendricks - current affiliation: Actual Labs Advisory (advisor and consultant), Vetted Capital (Venture Partner & Scientific Advisor), MI:RNA Diagnostics (Product Development Advisor); Krystal A. Orlando - current affiliation: National Institute of Environmental Health Sciences, National Institutes of Health; Elizabeth A. Raupach - current affiliation: Mayo Clinic in Arizona.

## STAR★Methods

### Key resources table

**Table:** 

REAGENT or RESOURCE	SOURCE	IDENTIFIER
**Antibodies**		

BRD4	Active Motif	Cat # 39909; RRID:AB_2793331
RPB1	Cell Signaling Technology	Cat # 2629; RRID:AB_2167468
c-MYC	Cell Signaling Technology	Cat # 2276; RRID:AB_331783
CyclinD3	Cell Signaling Technology	Cat # 2935; RRID:AB_1147658
PIM1	Cell Signaling Technology	Cat # 3247; RRID:AB_2299591
CDK6	Cell Signaling Technology	Cat # 3136; RRID:AB_2229289
SALL4	Abcam	Cat # Ab29112; RRID:AB_777810
Beta-actin	Cell Signaling Technology	Cat # 4970; RRID:AB_2223172
Beta-actin	ThermoFisher Scientific	Cat # MA5-15739; RRID:AB_10979409
Anti-rabbit IgG-HRP	Cell Signaling Technology	Cat # 7074; RRID:AB_2099233
Anti-mouse IgG-HRP	Cell Signaling Technology	Cat # 7076; RRID:AB_330924
IRDye 800CW Goat anti-Mouse	Li-Cor	Cat # 925-32210; RRID:AB_2687825
IRDye 800CW Goat anti-Rabbit	Li-Cor	Cat # 926-32211; RRID:AB_2651127
IRDye 680RD Goat anti-Rabbit	Li-Cor	Cat # 925-68071; RRID:AB_2721181
IRDye 680RD Goat anti-Mouse	Li-Cor	Cat # 926-68070; RRID:AB_2651128
SALL4 clone 6E3	Cell Marque	Cat # 385M
H3K27ac	Active Motif	Cat # 39133; RRID:AB_2561016
IgG (CUT&Tag)	Cell Signaling Technology	Cat # 2729; RRID:AB_1031062

**Biological samples**		

SCCOHT tumors	TGen biobank protocol, University of North Carolina biobank, University of British Columbia Cancer Agency biobank	https://www.tgen.org; https://www.bccrc.ca/services/biobanking-biospecimen-research-services-bbrs; https://bsp.web.unc.edu/
SCCOHT PDX tissue	Lang et al.^25^	Purchased from now inactive Molecular Response Laboratories

**Chemicals, peptides, and recombinant proteins**		

RIPA buffer	Santa Cruz Biotechnology	sc-24948
Triptolide	Cayman Chemical	11973
Minnelide	Minneamrita	N/A
CUTANA pAG-MNase	EpiCypher	15-1016

**Critical commercial assays**		

CellTiter-Glo	Promega	G753
ChIP DNA Clean & Concentrator	Zymo	D5205
D1000 Screen Tape	Agilent	5067-5584
D1000 Screen Tape Reagents	Agilent	5067-5585
Qubit dsDNA HS Assay Kit	ThermoFisher Scientific	Q32854
KAPA HyperPrep Kit	Roche	07962363001
NovaSeq SP 100 cycle kit	Illumina	20027464
Quick-DNA/RNA Miniprep Plus Kit	Zymo	D7003
KAPA mRNA HyperPrep Kit library prep kit	Roche	08098123702
NovaSeq S4 300 cycle kit	Illumina	20028312
RNeasy FFPE Kit	Qiagen	73504
SureSelect XT RNA	Agilent	G9691-90000

**Deposited data**		

H3K27ac CUT&RUN for BIN67, SCCOHT1, and COV434	GEO	GEO: GSE232727
H3K27ac CUT&RUN for BIN67 and COV434 treated with triptolide	GEO	GEO: GSE232745
RNA-seq for BIN67 and COV434 treated with triptolide	GEO	GEO: GSE232831
RNA-seq on SCCOHT tumors	dbGaP	dbGaP: phs001528.v2.p1
RNA-seq on SCCOHT tumors	GEO	GEO: GSE216801
RNA-seq on SCCOHT tumors	dbGaP	dbGaP: phs001528.v1.p1
RNA-seq on SCCOHT tumors	GEO	GEO: GSE109919

**Experimental models: Cell lines**		

BIN67	Barbara Vanderhyden lab; Upchurch et al.^36^	RRID:CVCL_S987
COV434	David Huntsman lab; Karnezis et al.^37^	RRID:CVCL_2010
SCCOHT-1	Ralf Hass lab; Otte et al.^38^	RRID:CVCL_VU69

**Experimental models: Organisms/strains**		

NOG (NOD.Cg-*Prkdc*^*scid*^*Il2rg*^*tm1Sug*^/JicTac) mice	Taconic	NOG-F

**Oligonucleotides**		

KAPA UDI Adapter	Roche	08861919702

**Software and algorithms**		

GraphPad Prism	GraphPad	https://www.graphpad.com/features
TGen Phoenix pipelines	TGen	https://github.com/tgen/phoenix
Rank Ordering of Super-Enhancers	Young Lab^20^^,^^21^	http://younglab.wi.mit.edu/super_enhancer_code.html
ChIPpeakAnno	Zhu et al.^39^ and Zhu^40^	https://bioconductor.org/packages/release/bioc/html/ChIPpeakAnno.html
csaw	Lun and Smyth^41^ and Lun and Smyth^42^	https://bioconductor.org/packages/release/bioc/html/csaw.html
DESeq2	Love et al.^43^	https://bioconductor.org/packages/release/bioc/html/DESeq2.html
Intervene	Khan and Mathelier^44^	https://intervene.readthedocs.io/en/latest/index.html
deepTools	Ramirez et al.^45^	https://deeptools.readthedocs.io/en/develop/
PANTHER v16.0	Mi et al.^46^	http://geneontology.org/

### Experimental model and study participant details

#### Cell lines

SCCOHT cell lines BIN67 and SCCOHT-1 were maintained in RPMI 1640 (Thermo Fisher Scientific) supplemented with 10% Fetal Bovine Serum (FBS; Thermo Fisher Scientific) and 1% Penicillin/Streptomycin (Thermo Fisher Scientific). COV434 cells were cultured in DMEM (Thermo Fisher Scientific) supplemented with 10% FBS and 1% Penicillin/Streptomycin. COV434 was previously identified as derived from a juvenile granulosa cell tumor but has now been re-categorized as SCCOHT based on SMARCA4 mutation, lack of SMARCA2 expression, and re-evaluation of original tumor specimen.^37^ All cells were maintained at 37°C in a humidified incubator containing 5% CO_2_ and were routinely monitored for mycoplasma testing (MycoSensor QPCR Assay Kit; Agilent) and STR profiled for cell line verification (IDEXX CellCheck). All cell line work was performed under TGen biosafety committee approval. All cell lines used in this study are derived from SCCOHT tumors from biological females. SCCOHT tumors only arise in biological females (gynecological cancer), and thus there is no sex differences to be addressed.

#### Animals

All procedures were carried out under the approval of Translational Drug Development’s Institutional Animal Care and Use Committee (IACUC protocol #19065). Histologically confirmed SMARCA4-mutant SCCOHT tumors PDX-465 and PDX-040 were acquired from Molecular Response (MRL) and serially passaged in female NOG (NOD.Cg-*Prkdc*^*scid*^ *Il2rg*^*tm1Sug*^/JicTac) mice (Taconic), starting at age 6-7 weeks, until tumors reached 1000 cubic mm or mice were 6 months old.^4^ Female mice were used for all studies, as SCCOHT ovarian cancers cannot occur in male mice, so this would be biologically irrelevant. Husbandry and housing follows standard IACUC procedures at Translational Drug Development (TD2).

#### Human participants

Frozen and formalin-fixed paraffin-embedded (FFPE) human SCCOHT specimens were obtained under TGen Western IRB protocol #1119451, University of North Carolina-Chapel Hill IRB protocol #90-0573, and University of British Columbia Cancer Agency REB project #H19-02823. For the tissue microarray (TMA), the University of Heidelberg ethics committee waived patient consent and approved the use of tissue samples under protocol S-463/19. All samples were deidentified and established to be non-human subjects research. Due to the large variance in consenting amongst the SCCOHT patients, clinical information associated with these patients will only be available for the patients eligible for uploading to dbGaP: phs001528.v2.p1. Patient demographics are available with the dbGaP metadata—due to the rare nature of this tumor type, we are unable to provide age ranges or ethnicity/ancestry/race without controlled access. SCCOHT tumors only arise in biological females (gynecological cancer), and thus there is no sex differences to be addressed. Twelve SCCOHT tumors were used for RNAseq—data sharing through dbGaP was only approved for ten tumors. A separate set of fourteen SCCOHT tumors available in a tissue microarray are included for SALL4 staining. There are no experimental groups to consider for this study.

### Method details

#### Drug dose response assay

SCCOHT cell lines BIN67, SCCOHT-1, and COV434 were each plated into 384-well plates at 1,000 cells per well and incubated at 37°C overnight. A 20-point, 3-fold serial dilution of triptolide ranging from 0.04 nM to 50 μM was applied to cells for 72 hours. CellTiter-Glo (Promega; Cat no. G753) was used per the manufacturer’s instructions to determine cell viability and read on a luminescent plate reader. 3-6 technical replicates were performed per experiment, and three independent replicates were performed for each cell line. The analysis is described in the quantification and statistical analysis section.

#### Western blotting

Whole-cell extracts from cell lines were prepared using RIPA buffer (Santa Cruz Biotechnology) with protease and phosphatase inhibitors or 9M urea buffer (9M urea, 4% CHAPS, 0.5% IPG buffer, 50mM DTT, all from Sigma Aldrich) using standard protocols. Thirty μg protein was loaded per well on NuPage 4-12% Bis-Tris gels or Tris Acetate gels (SALL4) and subsequently transferred to PVDF membranes.

For blots detected by chemiluminescence: Blots were pre-blocked in 5% non-fat dry milk in TBST or BSA in TBST for 1 hour and probed using primary antibody overnight. Blots were incubated with secondary antibody (anti-rabbit IgG-HRP, Cell Signaling Technology; anti-mouse IgG-HRP, Santa Cruz Biotechnology) at 1:5000 for 2 hours and developed using Pierce ECL Western Blotting Substrate or SuperSignal West Femto Substrate (Thermo Fisher Scientific).

For blots detected by fluorescence: Blots were blocked in Li-Cor Odyssey Blocking Buffer for 1 hour and probed using primary antibody overnight. Blots were incubated with secondary antibodies (IRDye 800CW Goat anti-Mouse #925-32210 or anti-Rabbit #926-32211 and IRDye 680RD Goat anti-Rabbit #925-68071 or anti-Mouse #926-68070, Li-Cor) at 1:20,000 for 2 hours and scanned on Li-Cor CLx.

Primary antibodies: BRD4 (Active Motif, #39909), RPB1 (Cell Signaling Technology, #2629), c-MYC (Cell Signaling Technology, #2276), CyclinD3 (Cell Signaling Technology, #2935), PIM1 (Cell Signaling Technology, #3247), CDK6 (Cell Signaling Technology, #3136), SALL4 (Abcam, ab29112), beta-actin (Cell Signaling Technology, #4970; or ThermoFisher Scientific MA5-15739).

#### Tissue microarrays and immunohistochemistry

Formalin-fixed paraffin-embedded tumor material from the TMA with 14 SCCOHT was stained using a mouse monoclonal SALL4 antibody (clone 6E3; RTU; Cell Marque, Rocklin, CA, USA) on a Ventana BenchMark XT immunostainer (Ventana Medical Systems, Tucson, AZ, USA) using standard protocols. SALL4 IHC scoring was performed according to the 3-tier system used by McCluggage et al.^31^ In brief, a score of 0 indicates no staining, 1 indicates <50% staining, and 2 indicates >50% staining.

#### Genomic library preparations and sequencing

CUT&RUN: DNA enriched in binding to H3K27acwt was determined using CUT&RUN and sequencing. Three independent replicates were performed for untreated CUT&RUN, and four independent replicates were performed for triptolide-treated cell lines. Cell lines were harvested at greater than 70% confluency with 0.05% trypsin. 10cm plates were seeded with 2-3x10^6^ BIN67 or COV434 cells and allowed to adhere overnight. The next day, fresh media was added with 100nM triptolide with a total DMSO concentration of less than 0.3%. After 6 or 16 hours of drug treatment, cells were harvested with 0.05% trypsin, and the total cell amount was determined with cell count with dead cell exclusion with trypan blue staining using a Countess II. For tissues, we used 10-20mg frozen tissues and disassociated them by pipetting in the first buffer in the protocol. EpiCypher CUTANA CUT&RUN protocol v1.5.2 was followed with 1x10^6^ cells used for input of each cell line for both the H3K27ac and control (IgG) sample. H3K27ac antibody (Active Motif, cat#39133) was used at a 1:50 dilution (1ug/sample) in the antibody buffer and incubated overnight at 4°C. A normal rabbit IgG antibody (CST, cat#2729) was similarly used at a 1:50 dilution (1ug/sample). For targeted DNA cleavage, 2μL of EpiCypher CUTANA pAG-MNase (cat#15-1016) was added to each reaction. After completing the CUT&RUN protocol, each sample was purified using the ChIP DNA Clean & Concentrator kit (ZYMO Research, cat#D5205). Samples were eluted in 30-50μL. The material was QC’d using an Agilent 4200 TapeStation with High Sensitivity D1000 ScreenTape (cat#5067-5584, reagents cat#5067-5585) and Qubit dsDNA HS Assay Kit (ThermoFisher, cat#Q32854). Typical yields were single-digit nanograms with the main DNA fragment detected at ∼150bp. KAPA HyperPrep Kit (Roche, cat#07962363001) was used to generate DNA libraries for sequencing. Protocol v6.17 was followed with the following modifications: (a) 1ng of input DNA from the CUT&RUN experiments, (b) 0.5μL of 1.5μM KAPA UDI Adapter (Roche, cat#08861919702) adapter stock was used during adapter ligation step, (c) final KAPA bead purification used a first cut of 0.65x beads followed by 0.25x bead purification. Samples were QC as above using TapeStation and Qubit. Typical library yields were ∼500ng. An equimolar pooling of libraries was performed for a final 1500pmols in 150μL with Tris pH8.5 buffer as the final fill volume. Pooled libraries were loaded onto a NovaSeq6000 using a NovaSeq SP 100 cycle kit (cat#20027464) (51x9x9x51). Basal H3K27ac on BIN67, SCCOHT1, and COV434 is available on GEO: GSE232727. H3K27ac on triptolide or vehicle-treated cells is also available on GEO: GSE232745.

RNA-seq: Vehicle- or triptolide-treated SCCOHT cell lines were harvested in parallel to the CUT&RUN samples described above. Four independent replicates were performed. RNA was extracted using Quick-DNA/RNA Miniprep Plus Kit (Zymo Research Cat#D7003), per manufacturer’s instructions, with on-column DNase digestion. 500ng of total RNA was used to generate mRNA libraries using the KAPA mRNA HyperPrep Kit library prep kit (Roche, cat#08098123702) per the manufacturer’s instructions. Final libraries were evaluated by TapeStation, quantitated using the Life Technologies/Invitrogen Qubit, and equimolarly pooled. Paired-end sequencing was performed on a NovaSeq6000 using a NovaSeq S4 300 cycle kit (cat#20028312) (151x11x11x151). RNAseq on triptolide or vehicle-treated cells is also available on GEO: GSE232831.

SCCOHT tumor RNA-seq was performed on a total of 10 tumors. Four tumors were previously described in Lang et al.^25^ (dbGaP: phs001528.v1.p1, GEO: GSE109919), which were sequenced to an average depth of 198.28 +/- 64.12 (standard deviation) million aligned reads. Six additional tumors were collected under IRB protocols: Western IRB protocol #1119451 at TGen (Samples TGEN-E through TGEN-I) and protocol #90-0573 at the University of North Carolina-Chapel Hill (Samples TGEN-J). This data is available at dbGaP: phs001528.v2.p1 and GEO: GSE216801. For the additional six tumors, RNA was harvested from formalin-fixed, paraffin-embedded (FFPE) SCCOHT tumors using the RNeasy FFPE Kit (Qiagen). Total RNA (200 ng) was heat fragmented on a GeneAmp PCR System 9700 (Applied Biosystems, Waltham, MA) to a target peak of 150 base pairs, and libraries were generated using the SureSelect XT RNA (Agilent) capture following the manufacturer’s protocol. RNA libraries were quantified, and quality was evaluated using the Qubit DNA BR Reagent kit and Tapestation D1000 tapes. Libraries were pooled by equimolar ratios and sequenced on a HiSeq4000 (Illumina) using paired-end sequencing (77x9x77). The average sequencing depth was 77.05 +/- 26.44 (standard deviation) million aligned reads.

#### Genomic data visualization

Tracks were visualized using either the UCSC Genome Browser or the Washington University Epigenome Browser. Tracks not from data in this study were acquired from the “ENCODE Regulation Layered H3K27ac Track,” available as a pre-loaded option on the UCSC Genome Browser (for 7 ENCODE cell lines). For expanded data in Epigenome Browser, the following datasets were used: ENCODE: ENCFF770KNQ, ENCFF854EEY, ENCFF668IIP, ENCFF028RHD, ENCFF587KMI, ENCFF724LRJ, ENCFF583PQT, ENCFF071XTD and GEO: GSE227779.

#### Public genomic datasets

Publicly available ChIP-seq data was also used to determine SWI/SNF binding at SEs and other genomic sites (GEO: GSE117735).^14^ The analysis is described in the quantification and statistical analysis section.

#### DepMap CRISPR analysis

Publicly available CRISPR data from the DepMap’s Project Achilles (Public 22Q4) were downloaded for BIN67, SCCOHT-1, and COV434 and the 30 identified SE-associated oncogenes from our independent SE analysis. See the quantification and statistical analysis section for details on the analysis.

#### Mouse drug efficacy study

Tumor suspension in 50% Matrigel / 50% Media in a final volume of 100 μL was subcutaneously inoculated into the backs of 6-7 week-old female NOG (NOD.Cg-*Prkdc*^*scid*^ *Il2rg*^*tm1Sug*^/JicTac) mice (Taconic). Mice were randomized to treatment arms (n=10) once the average tumor volume reached 75-125 mm^3^, when mice were 12 weeks old (minnelide study) or 15 weeks old (triptolide study). Triptolide (Cayman Chemical) was formulated in DMSO, and minnelide (Minneamrita Therapeutics) was formulated in PBS. Vehicle (DMSO or PBS), triptolide (0.6 mg/kg QD days 1-5, QOD days 10-60), or minnelide (0.42 mg/kg QD days 1-26) were administered by intraperitoneal injection (i.p.). Tumor size and body weight were measured twice weekly until the endpoint. Tumors were excised upon necropsy and either frozen or formalin-fixed and paraffin-embedded (FFPE).

### Quantification and statistical analysis

#### Drug dose response assay analysis

Background-subtracted luminescence readings were analyzed in GraphPad Prism with non-linear regression fit to calculate an IC_50_ value. The mean of technical replicates was calculated for independent experiments, and the means of each independent experiment was used to calculate the IC_50_ value.

#### Genomic analyses

CUT&RUN read alignment and peak calling were performed using modified ChIP-seq workflows implemented in TGen’s Phoenix pipeline (https://github.com/tgen/phoenix). First, BCL conversion was performed using Illumina’s Bcl Converter tool and parsing by barcode into independent FASTQ files. Alignment to GRCh38 human reference genome was performed using bowtie2 (v2.4.2)^47^ to generate BAM files (arguments: --local --very-sensitive-local --no-unal -- no-mixed --no-discordant --phred33 --minins 10 --maxins 700 -q), which were converted into CRAM format using samtools view -C. QC was assessed using FastQC (v0.11.8), samtools stats (v1.11),^48^ and deepTools (v3.3.1).^45^ Peaks were called using MACS2 callpeak (v2.2.6)^49^ with the following arguments: -f BAMPE -B -q 0.01 --keep-dup all. After the removal of peaks from the ENCODE Unified GRCh38 Exclusion List (blacklist; ENCODE: ENCFF356LFX), ROSE^20^^,^^21^ and CREAM^50^ were used to call SEs from the resulting narrowPeak files from MACS2. For ROSE, the ROSE_bedtoGFF.py was used to convert narrowPeak to GFF format, followed by ROSE_main.py (arguments: -g hg38 -s 12500 -5 2500). The R package CREAM tool was also used (arguments: MinLength = 1000, peakNumMin = 2). ROSE and CREAM SEs for each sample were concatenated together and merged using bedtools. Genome regions were mapped to genes using ChIPpeakAnno (v3.24.2) and ROSE_geneMapper.py (ROSE v0.1). Only ‘PROXIMAL_GENES’ were considered SE-associated genes to prevent the inclusion of H3K27ac signal overlapping promoters. Oncogenes from the merged set of genes were identified from the COSMIC tier-one cancer gene consensus list (v93, downloaded consensus table Jan 5, 2021). ChIPpeakAnno output was annotated with the R package EnsDb.Hsapiens.v86 (v2.99.0) and ROSE_geneMapper.py output were annotated using the ROSE gene reference table (hg19_refseq.ucsc). Differential peak calling was performed using csaw (v1.30.1, windowCounts width=150, pe=”both”) with DESeq2 (v1.36.0), calling differential peaks within the SEs called from independent H3K27ac CUT&RUN run on the same cell line. SE region intersections and upset plotting were performed with Intervene (v0.6.5,--bedtools-options f='.2').

Public data analysis was performed from downloaded SRA files that were converted to FASTQs. Alignment and peak calling were performed as above with the following modifications/additions to MACS2 callpeak: -f BAM -q 0.001 --SPMR. SWI/SNF peaks were defined by using bedtools intersect to find the overlap of SMARCC1 with DPF2 or ARID2 for BAF and PBAF peaks, respectively, which was performed separately on BIN67 control and BIN67 SMARCA4 WT re-expression. Peak summits defined all peak centers. All BAF and PBAF sites in control and SMARCA4 re-expression conditions were merged for the full set of SWI/SNF binding sites. IP enrichment at each of these sites was calculated using bedtools coverage with the resulting BED file of SWI/SNF binding sites as -a, and SMARCC1, H3K27ac, and Input in control and SMARCA4 WT re-expression BAM files as -b. The read counts were then normalized as follows: (total IP signal in the region per million mapped reads + pseudo count of 0.1)/(total input signal in the region per million mapped reads + pseudo count of 0.1). Annotation of SWI/SNF binding sites was performed using ChIPPeakAnno (v3.22.4)^39^^,^^40^ with built-in hg38 transcription start sites (data("TSS.human.GRCh38") and featureType = "TSS"). Proximal sites were defined as those that overlapped with TSSs, and Distal sites were those >2000kb from the nearest TSS. Distal sites were further assessed for falling within or outside SEs called from ROSE from the same dataset (merged from H3K27ac-derived SEs from control and SMARCA4 re-expression). Heatmaps were generated using deepTools with normalization with the RPKM method.

RNA-seq data was analyzed using RNA workflows implemented in TGen’s Phoenix pipeline (https://github.com/tgen/phoenix). Briefly, alignment of FASTQ files against the GRCh38 human reference genome was performed using STAR (v2.7.7a),^51^ and gene expression estimates were performed using Salmon (v1.4.0).^52^ Gene-level quantification in Transcripts Per Million kilobases (TPM) was used in subsequent steps. Where separate batches were analyzed together (as in cell line and tumor RNA-seq heatmaps), the batch correction was performed by first performing variance-stabilizing transformation, then using removeBatchEffect in the limma R package (v3.44.3).^53^ Differential expression analysis was performed in DESeq2 (v1.30.0)^43^ using cutoffs for log_2_FoldChange of +/- 1.5 and adjusted p-value < 0.05 to determine significance. Heatmaps were generated using pheatmap in R using the 'complete' clustering method and Euclidean distances. For input to gene ontology analysis, SE-associated genes in the top 2 high expressing clusters for gene expression (Figure 2C, purple box; Table S3) were input into PANTHER v16.0 (http://geneontology.org/), and terms with FDR ≤ 0.05 were visualized. For comparison of SCCOHT data to TCGA and ENCODE normal ovary and reference cell lines, FPKM expression values were used.

#### DepMap CRISPR analysis

The mean Chronos score of the three cell lines was used for plotting against the average change in gene expression following triptolide treatment (see above) using GraphPad Prism. Data presented in Figure 5D represents mean +/- SD.

#### Other statistical analyses

The chi-square test was conducted using built-in functions in Excel for the table in Figure 4E. Statistical tests for animal tumor weights were performed in GraphPad Prism using unpaired t-tests with multiple comparison testing using the two-stage step-up method, which combines Benjamini, Krieger, and Yekutieli methods for multi-timepoint analyses (Figures S6A, S6C, 6E, and 6F). Unpaired t-tests were used for single-timepoint statistical tests (Figures 6B, 6D, S6C, and S6D).

## Supplemental information

Document S1. Figures S1–S6 and Table S3

Table S1. Table of gene ontology results

Table S2. Table of differential gene expression with triptolide treatment

## References

[1] Witkowski L., Goudie C., Ramos P., Boshari T., Brunet J.-S., Karnezis A.N., Longy M., Knost J.A., Saloustros E., McCluggage W.G. (2016). The influence of clinical and genetic factors on patient outcome in small cell carcinoma of the ovary, hypercalcemic type. Gynecol. Oncol..

[2] Tischkowitz M., Huang S., Banerjee S., Hague J., Hendricks W.P.D., Huntsman D.G., Lang J.D., Orlando K.A., Oza A.M., Pautier P. (2020). Small-Cell Carcinoma of the Ovary, Hypercalcemic Type-Genetics, New Treatment Targets, and Current Management Guidelines. Clin. Cancer Res..

[3] Witkowski L., Carrot-Zhang J., Albrecht S., Fahiminiya S., Hamel N., Tomiak E., Grynspan D., Saloustros E., Nadaf J., Rivera B. (2014). Germline and somatic SMARCA4 mutations characterize small cell carcinoma of the ovary, hypercalcemic type. Nat. Genet..

[4] Karnezis A.N., Wang Y., Ramos P., Hendricks W.P., Oliva E., D’Angelo E., Prat J., Nucci M.R., Nielsen T.O., Chow C. (2016). Dual loss of the SWI/SNF complex ATPases SMARCA4/BRG1 and SMARCA2/BRM is highly sensitive and specific for small cell carcinoma of the ovary, hypercalcaemic type. J. Pathol..

[5] Ramos P., Karnezis A.N., Hendricks W.P.D., Wang Y., Tembe W., Zismann V.L., Legendre C., Liang W.S., Russell M.L., Craig D.W. (2014). Loss of the tumor suppressor SMARCA4 in small cell carcinoma of the ovary, hypercalcemic type (SCCOHT). Rare Dis..

[6] Jelinic P., Mueller J.J., Olvera N., Dao F., Scott S.N., Shah R., Gao J., Schultz N., Gonen M., Soslow R.A. (2014). Recurrent SMARCA4 mutations in small cell carcinoma of the ovary. Nat. Genet..

[7] Ramos P., Karnezis A.N., Craig D.W., Sekulic A., Russell M.L., Hendricks W.P.D., Corneveaux J.J., Barrett M.T., Shumansky K., Yang Y. (2014). Small cell carcinoma of the ovary, hypercalcemic type, displays frequent inactivating germline and somatic mutations in SMARCA4. Nat. Genet..

[8] Lin D.I., Chudnovsky Y., Duggan B., Zajchowski D., Greenbowe J., Ross J.S., Gay L.M., Ali S.M., Elvin J.A. (2017). Comprehensive genomic profiling reveals inactivating SMARCA4 mutations and low tumor mutational burden in small cell carcinoma of the ovary, hypercalcemic-type. Gynecol. Oncol..

[9] Chan-Penebre E., Armstrong K., Drew A., Grassian A.R., Feldman I., Knutson S.K., Kuplast-Barr K., Roche M., Campbell J., Ho P. (2017). Selective Killing of SMARCA2- and SMARCA4-deficient Small Cell Carcinoma of the Ovary, Hypercalcemic Type Cells by Inhibition of EZH2: In Vitro and In Vivo Preclinical Models. Mol. Cancer Therapeut..

[10] Wang Y., Chen S.Y., Karnezis A.N., Colborne S., Santos N.D., Lang J.D., Hendricks W.P., Orlando K.A., Yap D., Kommoss F. (2017). The histone methyltransferase EZH2 is a therapeutic target in small cell carcinoma of the ovary, hypercalcaemic type. J. Pathol..

[11] Wang Y., Chen S.Y., Colborne S., Lambert G., Shin C.Y., Santos N.D., Orlando K.A., Lang J.D., Hendricks W.P.D., Bally M.B. (2018). Histone Deacetylase Inhibitors Synergize with Catalytic Inhibitors of EZH2 to Exhibit Antitumor Activity in Small Cell Carcinoma of the Ovary, Hypercalcemic Type. Mol. Cancer Therapeut..

[12] Alver B.H., Kim K.H., Lu P., Wang X., Manchester H.E., Wang W., Haswell J.R., Park P.J., Roberts C.W.M. (2017). The SWI/SNF chromatin remodelling complex is required for maintenance of lineage specific enhancers. Nat. Commun..

[13] Jones C.A., Tansey W.P., Weissmiller A.M. (2022). Emerging Themes in Mechanisms of Tumorigenesis by SWI/SNF Subunit Mutation. Epigenet. Insights.

[14] Pan J., McKenzie Z.M., D’Avino A.R., Mashtalir N., Lareau C.A., St Pierre R., Wang L., Shilatifard A., Kadoch C. (2019). The ATPase module of mammalian SWI/SNF family complexes mediates subcomplex identity and catalytic activity-independent genomic targeting. Nat. Genet..

[15] Wang X., Lee R.S., Alver B.H., Haswell J.R., Wang S., Mieczkowski J., Drier Y., Gillespie S.M., Archer T.C., Wu J.N. (2017). SMARCB1-mediated SWI/SNF complex function is essential for enhancer regulation. Nat. Genet..

[16] Sengupta S., George R.E. (2017). Super-Enhancer-Driven Transcriptional Dependencies in Cancer. Trends Cancer.

[17] Hnisz D., Abraham B.J., Lee T.I., Lau A., Saint-André V., Sigova A.A., Hoke H.A., Young R.A. (2013). Super-Enhancers in the Control of Cell Identity and Disease. Cell.

[18] Shorstova T., Marques M., Su J., Johnston J., Kleinman C.L., Hamel N., Huang S., Alaoui-Jamali M.A., Foulkes W.D., Witcher M. (2019). SWI/SNF-Compromised Cancers Are Susceptible to Bromodomain Inhibitors. Cancer Res..

[19] Berns K., Caumanns J.J., Hijmans E.M., Gennissen A.M.C., Severson T.M., Evers B., Wisman G.B.A., Jan Meersma G., Lieftink C., Beijersbergen R.L. (2018). ARID1A mutation sensitizes most ovarian clear cell carcinomas to BET inhibitors. Oncogene.

[20] Whyte W.A., Orlando D.A., Hnisz D., Abraham B.J., Lin C.Y., Kagey M.H., Rahl P.B., Lee T.I., Young R.A. (2013). Master Transcription Factors and Mediator Establish Super-Enhancers at Key Cell Identity Genes. Cell.

[21] Lovén J., Hoke H.A., Lin C.Y., Lau A., Orlando D.A., Vakoc C.R., Bradner J.E., Lee T.I., Young R.A. (2013). Selective inhibition of tumor oncogenes by disruption of super-enhancers. Cell.

[22] Titov D.V., Gilman B., He Q.-L., Bhat S., Low W.-K., Dang Y., Smeaton M., Demain A.L., Miller P.S., Kugel J.F. (2011). XPB, a subunit of TFIIH, is a target of the natural product triptolide. Nat. Chem. Biol..

[23] Kwiatkowski N., Zhang T., Rahl P.B., Abraham B.J., Reddy J., Ficarro S.B., Dastur A., Amzallag A., Ramaswamy S., Tesar B. (2014). Targeting transcription regulation in cancer with a covalent CDK7 inhibitor. Nature.

[24] Xue Y., Meehan B., Macdonald E., Venneti S., Wang X.Q.D., Witkowski L., Jelinic P., Kong T., Martinez D., Morin G. (2019). CDK4/6 inhibitors target SMARCA4-determined cyclin D1 deficiency in hypercalcemic small cell carcinoma of the ovary. Nat. Commun..

[25] Lang J.D., Hendricks W.P.D., Orlando K.A., Yin H., Kiefer J., Ramos P., Sharma R., Pirrotte P., Raupach E.A., Sereduk C. (2018). Ponatinib Shows Potent Antitumor Activity in Small Cell Carcinoma of the Ovary Hypercalcemic Type (SCCOHT) through Multikinase Inhibition. Clin. Cancer Res..

[26] Carugo A., Minelli R., Sapio L., Soeung M., Carbone F., Robinson F.S., Tepper J., Chen Z., Lovisa S., Svelto M. (2019). p53 Is a Master Regulator of Proteostasis in SMARCB1-Deficient Malignant Rhabdoid Tumors. Cancer Cell.

[27] Howard T.P., Arnoff T.E., Song M.R., Giacomelli A.O., Wang X., Hong A.L., Dharia N.V., Wang S., Vazquez F., Pham M.-T. (2019). MDM2 and MDM4 Are Therapeutic Vulnerabilities in Malignant Rhabdoid Tumors. Cancer Res..

[28] Banerjee S., Saluja A. (2015). Minnelide, a novel drug for pancreatic and liver cancer. Pancreatology.

[29] Kroemer M., Spehner L., Mercier-Letondal P., Boullerot L., Kim S., Jary M., Galaine J., Picard E., Ferrand C., Nguyen T. (2018). SALL4 oncogene is an immunogenic antigen presented in various HLA-DR contexts. OncoImmunology.

[30] Jelinic P., Ricca J., Van Oudenhove E., Olvera N., Merghoub T., Levine D.A., Zamarin D. (2018). Immune-Active Microenvironment in Small Cell Carcinoma of the Ovary, Hypercalcemic Type: Rationale for Immune Checkpoint Blockade. J. Natl. Cancer Inst..

[31] McCluggage W.G., Witkowski L., Clarke B.A., Foulkes W.D. (2017). Clinical, morphological and immunohistochemical evidence that small-cell carcinoma of the ovary of hypercalcaemic type (SCCOHT) may be a primitive germ-cell neoplasm. Histopathology.

[32] Raupach E.A., Garcia-Mansfield K., Sharma R., Hegde A.M., David-Dirgo V., Wang Y., Shin C.Y., Tao L.V., Facista S.J., Moore R. (2019). Novel functional insights revealed by distinct protein-protein interactions of the residual SWI/SNF complex in SMARCA4-deficient small cell carcinoma of the ovary, hypercalcemic type. Cancer Biol..

[33] Noel P., Hussein S., Ng S., Antal C.E., Lin W., Rodela E., Delgado P., Naveed S., Downes M., Lin Y. (2020). Triptolide targets super-enhancer networks in pancreatic cancer cells and cancer-associated fibroblasts. Oncogenesis.

[34] Gorrie D., Bravo M., Fan L. (2024). The Yin and Yang of the Natural Product Triptolide and Its Interactions with XPB, an Essential Protein for Gene Expression and DNA Repair. Genes.

[35] Noel P., Von Hoff D.D., Saluja A.K., Velagapudi M., Borazanci E., Han H. (2019). Triptolide and Its Derivatives as Cancer Therapies. Trends Pharmacol. Sci..

[36] Upchurch K.S., Parker L.M., Scully R.E., Krane S.M. (1986). Differential cyclic AMP responses to calcitonin among human ovarian carcinoma cell lines: A calcitonin-responsive line derived from a rare tumor type. J. Bone Miner. Res..

[37] Karnezis A.N., Chen S.Y., Chow C., Yang W., Hendricks W.P.D., Ramos P., Briones N., Mes-Masson A.-M., Bosse T., Gilks C.B. (2021). Re-assigning the histologic identities of COV434 and TOV-112D ovarian cancer cell lines. Gynecol. Oncol..

[38] Otte A., Göhring G., Steinemann D., Schlegelberger B., Groos S., Länger F., Kreipe H.-H., Schambach A., Neumann T., Hillemanns P. (2012). A tumor-derived population (SCCOHT-1) as cellular model for a small cell ovarian carcinoma of the hypercalcemic type. Int. J. Oncol..

[39] Zhu L.J., Gazin C., Lawson N.D., Pagès H., Lin S.M., Lapointe D.S., Green M.R. (2010). ChIPpeakAnno: a Bioconductor package to annotate ChIP-seq and ChIP-chip data. BMC Bioinf..

[40] Zhu L.J. (2013). Integrative analysis of ChIP-chip and ChIP-seq dataset. Methods Mol. Biol..

[41] Lun A.T.L., Smyth G.K. (2016). csaw: a Bioconductor package for differential binding analysis of ChIP-seq data using sliding windows. Nucleic Acids Res..

[42] Lun A.T.L., Smyth G.K. (2014). De novo detection of differentially bound regions for ChIP-seq data using peaks and windows: controlling error rates correctly. Nucleic Acids Res..

[43] Love M.I., Huber W., Anders S. (2014). Moderated estimation of fold change and dispersion for RNA-seq data with DESeq2. Genome Biol..

[44] Khan A., Mathelier A. (2017). Intervene: a tool for intersection and visualization of multiple gene or genomic region sets. BMC Bioinf..

[45] Ramírez F., Ryan D.P., Grüning B., Bhardwaj V., Kilpert F., Richter A.S., Heyne S., Dündar F., Manke T. (2016). deepTools2: a next generation web server for deep-sequencing data analysis. Nucleic Acids Res..

[46] Mi H., Ebert D., Muruganujan A., Mills C., Albou L.-P., Mushayamaha T., Thomas P.D. (2021). PANTHER version 16: a revised family classification, tree-based classification tool, enhancer regions and extensive API. Nucleic Acids Res..

[47] Langmead B., Salzberg S.L. (2012). Fast gapped-read alignment with Bowtie 2. Nat. Methods.

[48] Danecek P., Bonfield J.K., Liddle J., Marshall J., Ohan V., Pollard M.O., Whitwham A., Keane T., McCarthy S.A., Davies R.M., Li H. (2021). Twelve years of SAMtools and BCFtools. GigaScience.

[49] Zhang Y., Liu T., Meyer C.A., Eeckhoute J., Johnson D.S., Bernstein B.E., Nusbaum C., Myers R.M., Brown M., Li W., Liu X.S. (2008). Model-based analysis of ChIP-Seq (MACS). Genome Biol..

[50] Madani Tonekaboni S.A., Mazrooei P., Kofia V., Haibe-Kains B., Lupien M. (2019). Identifying clusters of cis-regulatory elements underpinning TAD structures and lineage-specific regulatory networks. Genome Res..

[51] Dobin A., Davis C.A., Schlesinger F., Drenkow J., Zaleski C., Jha S., Batut P., Chaisson M., Gingeras T.R. (2013). STAR: ultrafast universal RNA-seq aligner. Bioinformatics.

[52] Patro R., Duggal G., Love M.I., Irizarry R.A., Kingsford C. (2017). Salmon provides fast and bias-aware quantification of transcript expression. Nat. Methods.

[53] Ritchie M.E., Phipson B., Wu D., Hu Y., Law C.W., Shi W., Smyth G.K. (2015). limma powers differential expression analyses for RNA-sequencing and microarray studies. Nucleic Acids Res..

