# Supplementary material for: Super-enhancers and efficacy of triptolide in small cell carcinoma of the ovary hypercalcemic type
Source: iScience. 2025 Jan 7;28(2):111770. doi: 10.1016/j.isci.2025.111770 (PMC11791298; doi:10.1016/j.isci.2025.111770)
Supplement: Document S1. Figures S1–S6 and Table S3 [file mmc1.pdf]

iScience, Volume *28*

Supplemental information

Super-enhancers and efficacy of triptolide in small cell carcinoma of the ovary hypercalcemic type

Jessica D. Lang, William Selleck, Shawn Striker, Nicolle A. Hipschman, Rochelle Kofman, Anthony N. Karnezis, Felix K.F. Kommoss, Friedrich Kommoss, Jae Rim Wendt, Salvatore J. Facista, William P.D. Hendricks, Krystal A. Orlando, Patrick Pirrotte, Elizabeth A. Raupach, Victoria L. Zismann, Yemin Wang, David G. Huntsman, Bernard E. Weissman, and Jeffrey M. Trent

Supplemental data and table titles

Document S1. Figures S1-S6

Table S1. Table of gene ontology results

Table S2. Table of differential gene expression with triptolide treatment

Table S3. List of SE-associated oncogenes

Supplemental data and table legends

Figure S1. ChIP-seq and ATAC-seq signal heatmaps. (A) SWI/SNF occupancy sites from BIN67 cells (ChIP data re-analyzed from Pan *et al.*[14]) are annotated as proximal (overlapping TSS) or distal (>2kb) to TSS. Distal sites are separated by those overlapping SE sites (In SE), as determined by ROSE on H3K27ac ChIP data, and those non-overlapping those sites (Outside SE). Signal intensity over a 3kb region centered on the SWI/SNF peak center is shown with higher normalized read count in dark blue vs. white for absent read count. SMARCC1, DPF2, ARID2, and SMARCA4 ChIP-seq and ChIP-seq Input enrichment are plotted on the same scale. Due to different read depth for H3K27ac ChIP-seq and ATAC-seq, these each had different scale maximums from the SWI/SNF subunits, but Control and SMARCA4 re-expression conditions are on the same relative scale for all samples. (B) ATAC-seq signal at SWI/SNF occupancy sites, categorized by change of H3K27ac at the same site with SMARCA4 re-expression. A cutoff of > |log_2FoldChange in H3K27ac occupancy| was used to distinguish decreased/increased peaks from static peaks. A summary of the average of peak intensity is shown to the right of heatmaps, where the dotted line represents SMARCA4 re-expression. (C) Total number of SWI/SNF occupancy peaks by location annotation (x-axis) and H3K27ac change after SMARCA4 re-expression (stacked bar chart). (D) Percent of SWI/SNF occupancy peaks by location annotation.

Figure S2. SCCOHT SE-associated oncogene expression compared to other ovarian cancers and normal samples. In addition to Figure 3A, the remaining SE-associated oncogenes' RNA expression levels. RNA expression from RNA-seq on SCCOHT tumors, age-matched TCGA high-grade serous ovarian carcinoma (HGSC) tumors, normal ovary tissue from ENCODE (ENCODE ovary), and ENCODE reference cell lines (ENCODE reference). FPKM + 0.01 is plotted on a logarithmic scale, with individual circles representing each sample and the box and whiskers plot summarizing the median value (heavy line), upper and lower 25% quartiles (box limits), and error bars showing the highest and lowest values.

Figure S3. *SALL4* super-enhancer in expanded sample comparison. H3K27ac tracks at *SALL4* SE are shown in Figure 3B. The gray boxes indicate the common SE regions between the 3 SCCOHT cell lines. The H3K27ac ChIP-seq tracks were acquired from ENCODE and GEO datasets and loaded into the Epigenome Browser.

Figure S4. SALL4 expression in SCCOHT tumors. SCCOHT TMAs stained for SALL4. The patient number is indicated to the left of the core images. Each tumor sample has at least one set of two representative cores from a given area, which are positioned next to each other in the row. Patients with more than one pair of cores are presented in separate rows. The staining score for each sample pair is indicated to the right.

Figure S5. Differential occupancy analysis within SCCOHT SEs. Differential occupancy analysis was performed with csaw to identify peaks within BIN67 (A-B) and COV434 (C-D) SEs that change with triptolide treatment for 6 hours (A & C; N=4) and 16 hours (B & D, N=3). Cutoffs of | $\log_2$ fold change in peak signal | > 1.5 and false discovery rate of > 0.05 were used.

Figure S6. Triptolide and minnelide are well tolerated in mice bearing PDXs. (A) Individual tumor weights of SCCOHT PDX-040 treated with vehicle (DMSO i.p.; N=10) or triptolide (0.6 mg/kg i.p., QD days 1-5, QOD days 10-60, N=4). (B) Individual tumor weights of SCCOHT PDX-465 treated with vehicle (PBS i.p., N=10) or minnelide (0.42 mg/kg i.p., QD, N=10). Individual mouse measurements are shown in light lines. The group mean and standard error of the mean are shown in bold lines. (C) Individual body weights of mice bearing PDX-040 treated with triptolide. (D) Individual body weights of mice bearing PDX-465 treated with minnelide. (E) SCCOHT PDX-040 and (F) PDX-465 tumor weights were equivalent between vehicle and treatment groups at the study start. Data is represented as mean +/- SEM throughout.

Table S1. Gene ontology results for SE-associated genes. Gene ontology output details for data are summarized in Figure 2E.

Table S2. Differential expression analysis for BIN67 and COV434 cell lines treated with triptolide. DESeq2 output for data associated with Figure 4D & E and 5A-C.

Table S3. SE-associated genes in the top 2 high expressing clusters for gene expression.

Supplemental Figures

Figure S1

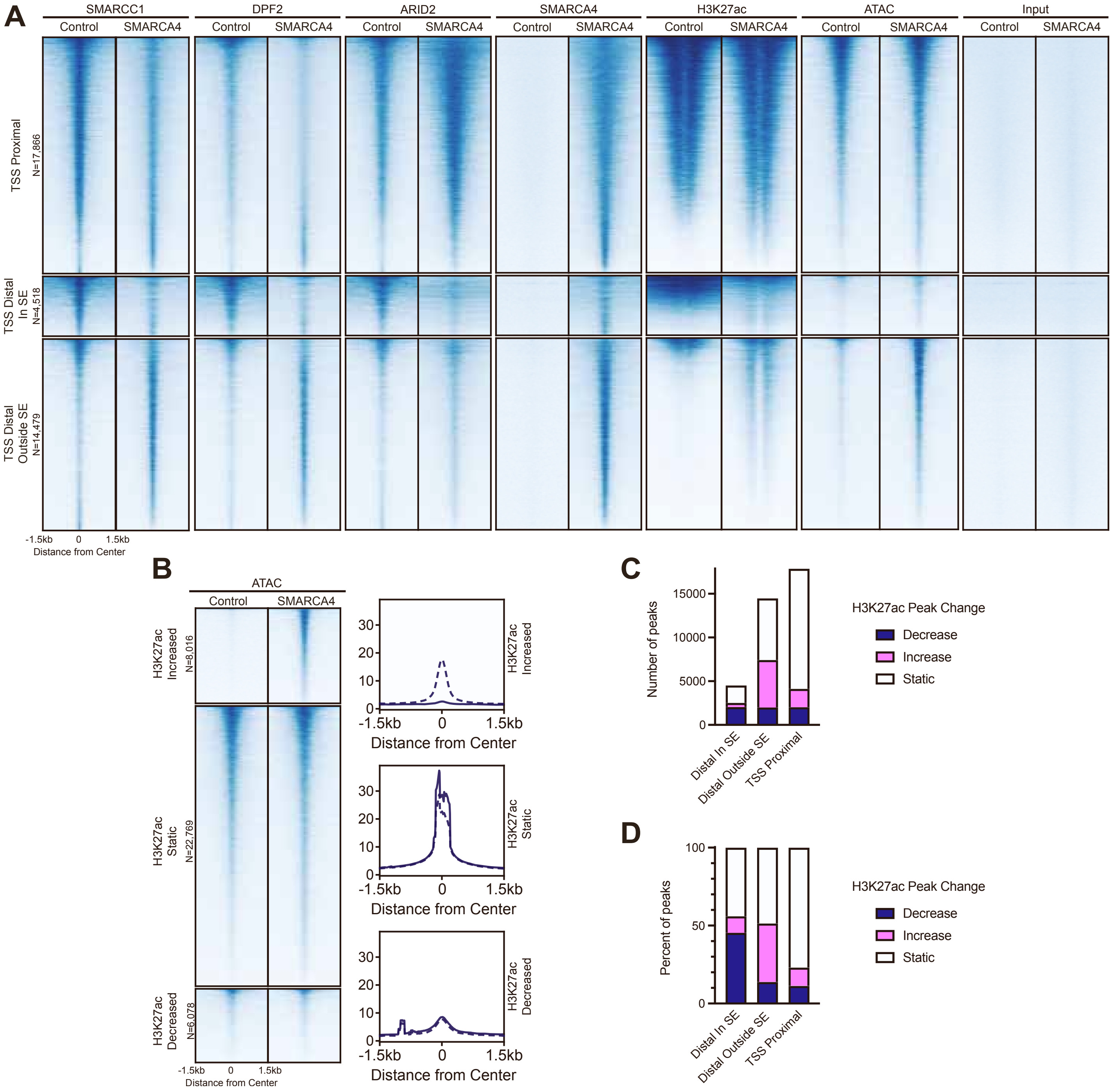

Figure S2

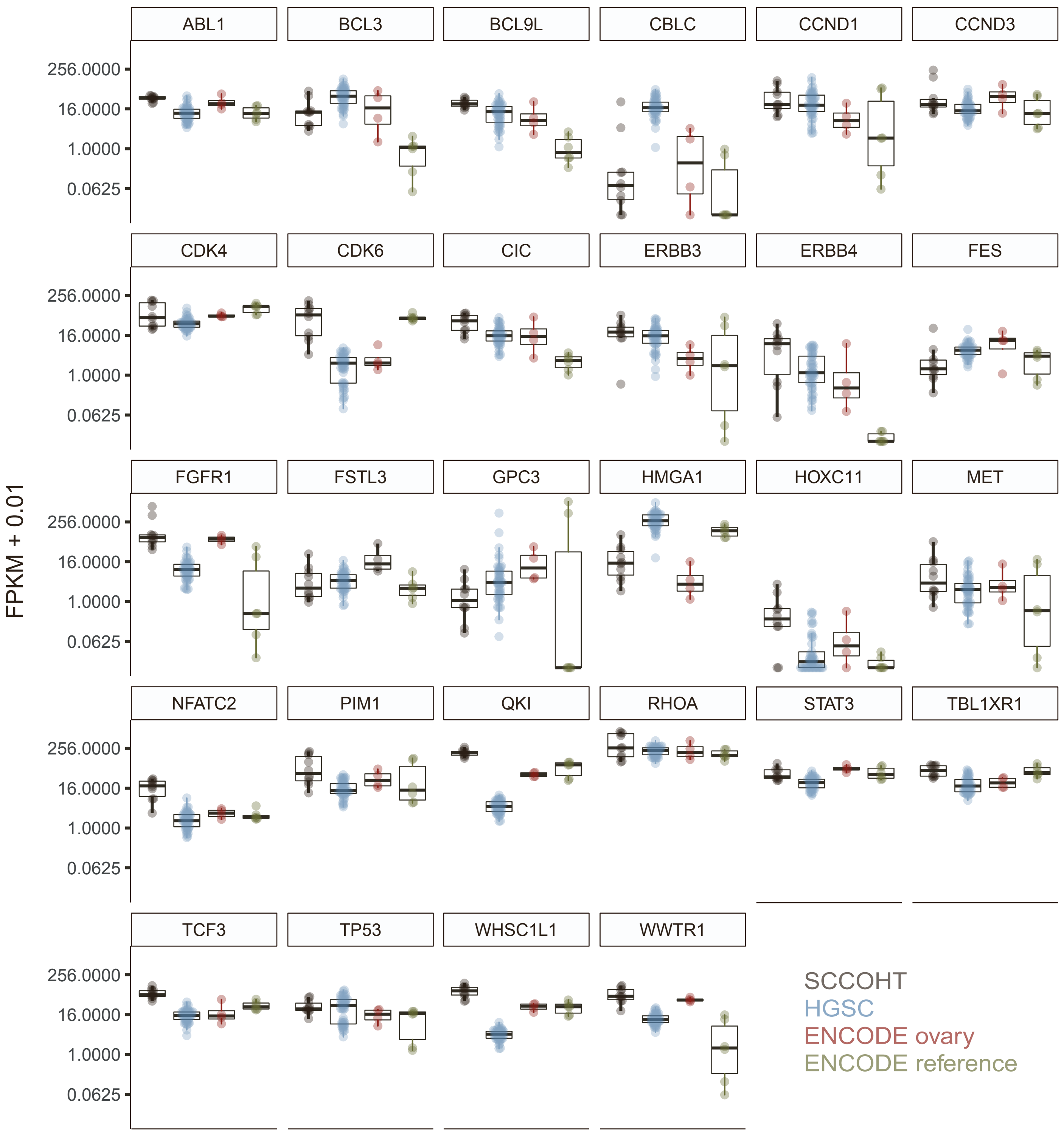

ABL1
BCL3
BCL9L
CBLC
CCND1
CCND3
CDK4
CDK6
CIC
ERBB3
ERBB4
FES
FGFR1
FSTL3
GPC3
HMGA1
HOXC11
MET
NFATC2
PIM1
QKI
RHOA
STAT3
TBL1XR1
TCF3
TP53
WHSC1L1
WWTR1
256.0000
16.0000
1.0000
0.0625
FPKM + 0.01
SCCOHT
HGSC
ENCODE ovary
ENCODE reference

Figure S3

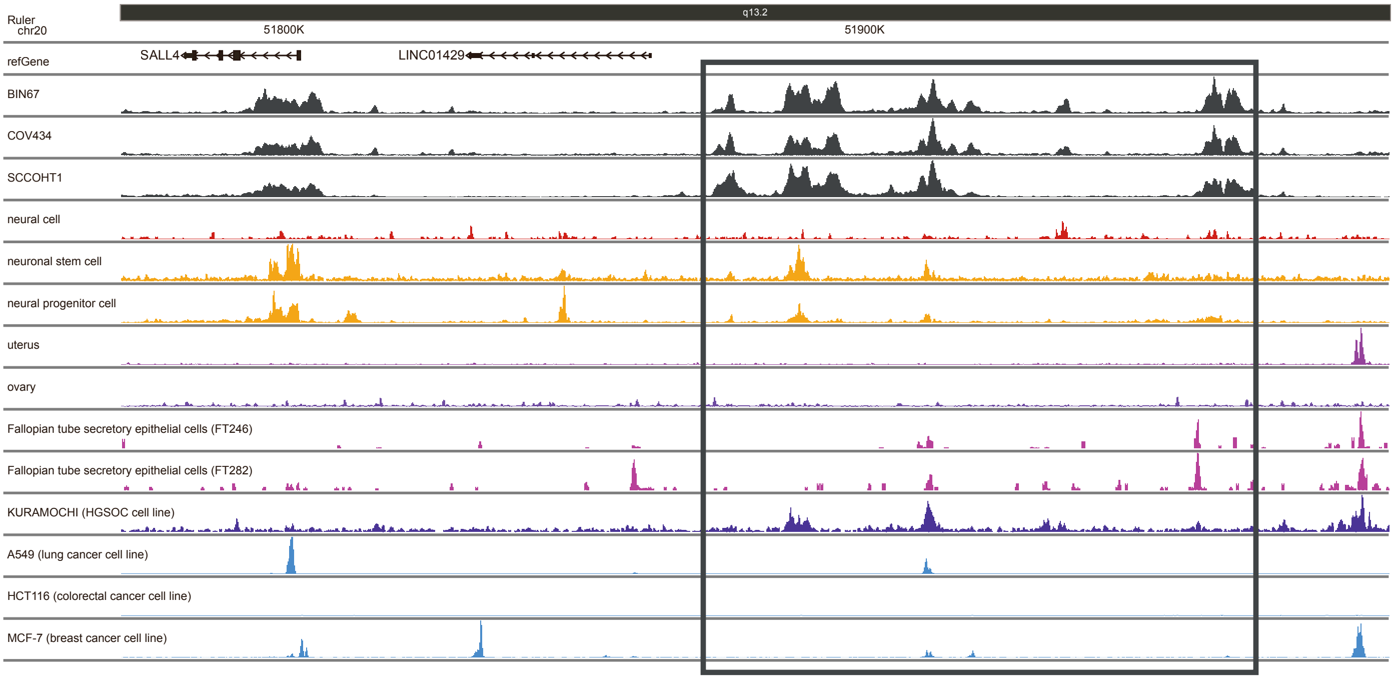
Ruler
chr20
q13.2
51800K
51900K
refGene
SALL4
LINC01429
BIN67
COV434
SCCOHT1
neural cell
neuronal stem cell
neural progenitor cell
uterus
ovary
Fallopian tube secretory epithelial cells (FT246)
Fallopian tube secretory epithelial cells (FT282)
KURAMOCHI (HGSOC cell line)
A549 (lung cancer cell line)
HCT116 (colorectal cancer cell line)
MCF-7 (breast cancer cell line)

Figure S4

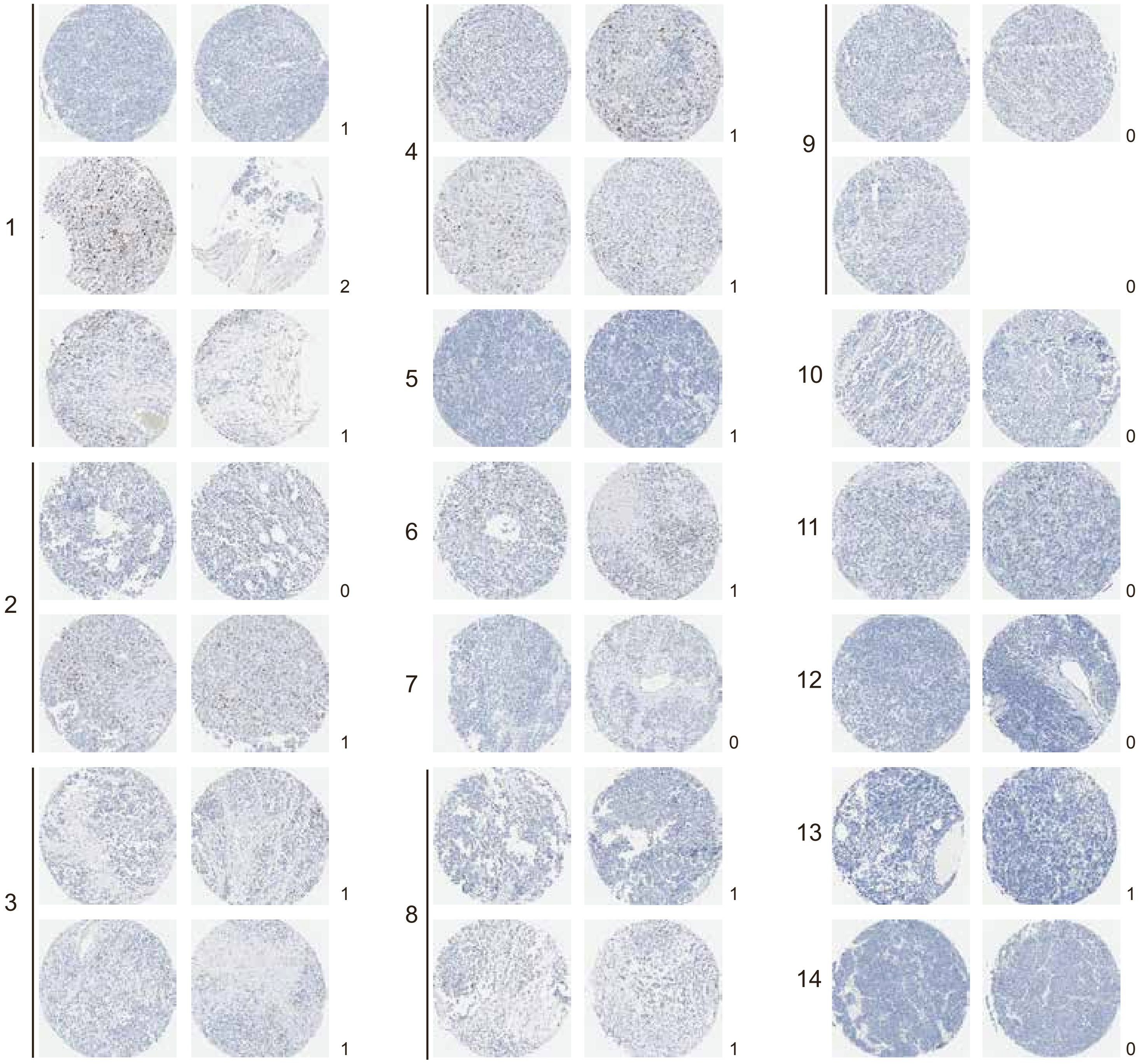

Figure S5

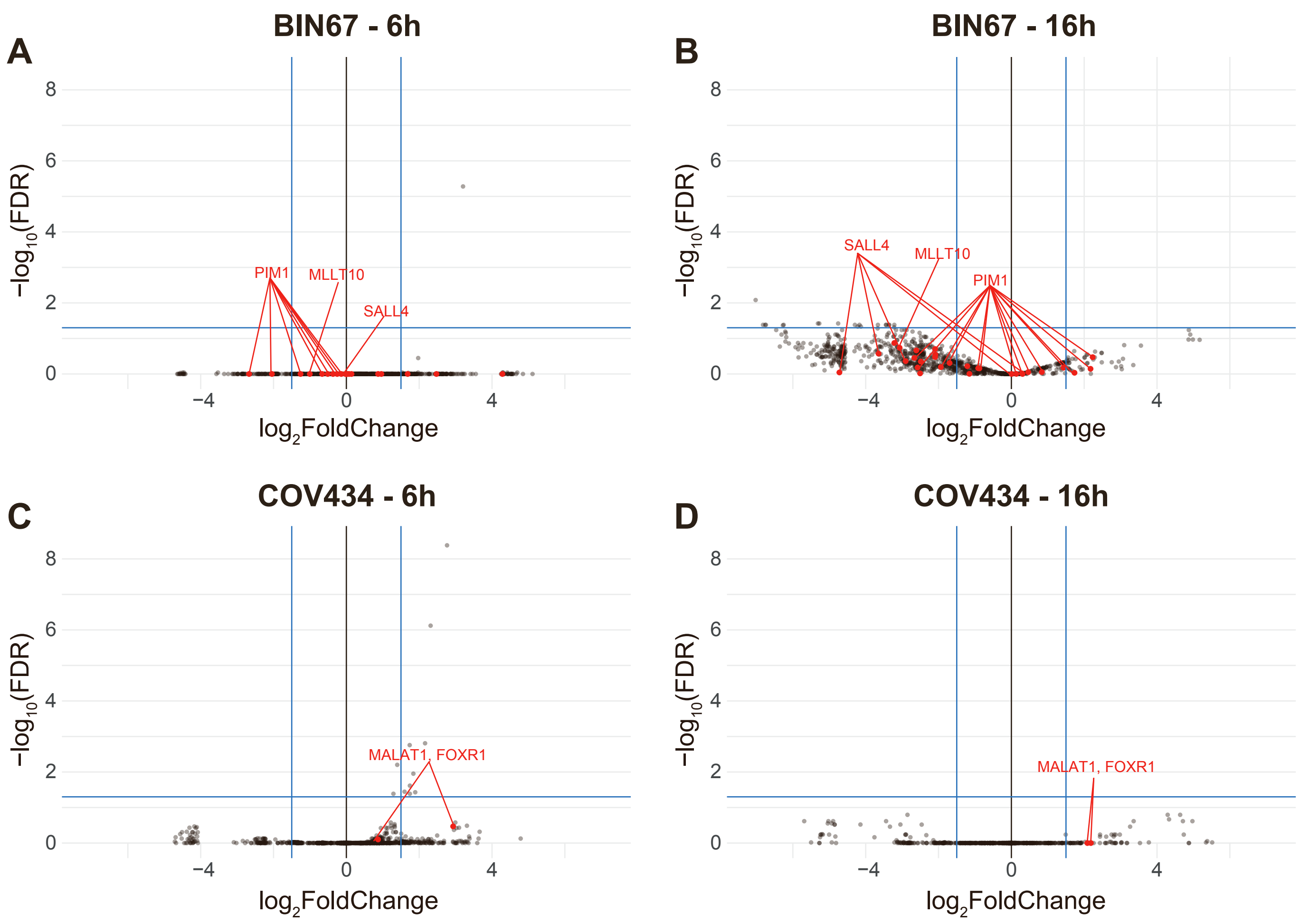

A
BIN67 - 6h
−log10(FDR)
log2FoldChange
PIM1
MLLT10
SALL4
B
BIN67 - 16h
−log10(FDR)
log2FoldChange
SALL4
MLLT10
PIM1
C
COV434 - 6h
−log10(FDR)
log2FoldChange
MALAT1, FOXR1
D
COV434 - 16h
−log10(FDR)
log2FoldChange
MALAT1, FOXR1

Figure S6

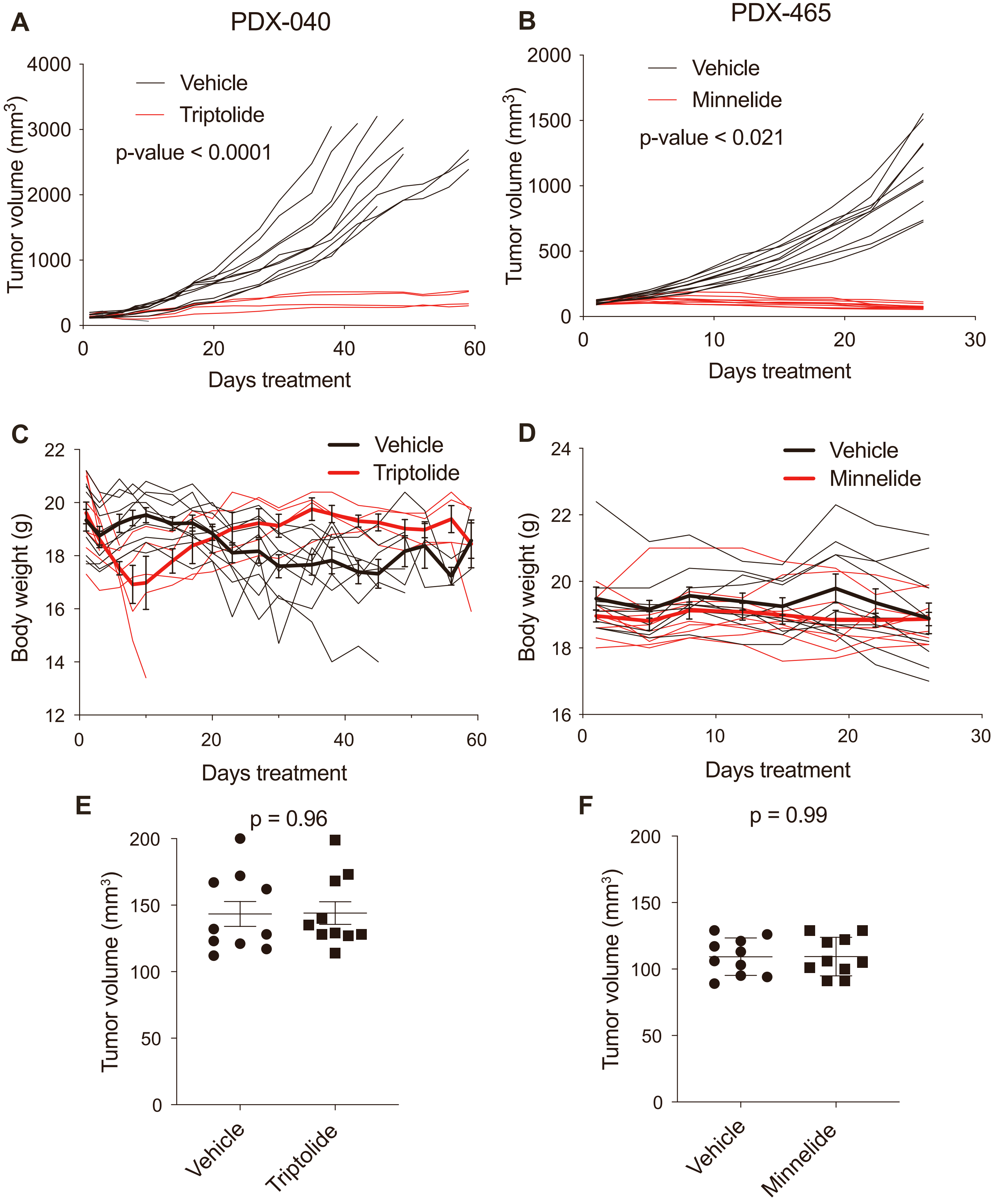
A
PDX-040
Vehicle
Triptolide
p-value < 0.0001
Tumor volume (mm^3)
Days treatment
B
PDX-465
Vehicle
Minnelide
p-value < 0.021
Tumor volume (mm^3)
Days treatment
C
Vehicle
Triptolide
Body weight (g)
Days treatment
D
Vehicle
Minnelide
Body weight (g)
Days treatment
E
p = 0.96
Tumor volume (mm^3)
Vehicle
Triptolide
F
p = 0.99
Tumor volume (mm^3)
Vehicle
Minnelide

Table S3

Gene Name
ACTB
MALAT1
SEMA3A
SGK1
EMP1
TES
CAV2
MID1
ID4
MDM1
DNM3OS
NR2F1
GAB2
LIMS1
USP25
AZIN1
IRF2BPL
MLLT10
STAU2
ATAD2B
RAPGEF2
DOCK7
STAT3
NEAT1
RAP1B
PLEKHG1
ARID5B
ADGRG6
MTATP6P1
SKIDA1
MIR1915HG
MEX3A
USP37
KMT2B
RAI1
ERF
FAM172A
GCC2
GABARAP
TRIR
ENSA
EMC10

IMPDH2
KDELR1
SETD1B
KDM6B
RNF216
ARIH2
MIR99AHG
CA11
BCL9L
NXF1
ATP11A
PLD3
ZC3H15
WDR75
LMNA
FSCN1
MTMR2
ERCC1
PAFAH1B3
GSK3A
CTDNEP1
LTBP3
SSBP4
EMP3
RHBDD1
GET3
ZFP64
PTPN9
CTDSP1
VASP
MAN2A2
TRMT112
PRDX5
SCARB1
OTUD7B
CYTH2
CARD8
PRCC
DVL2
TNRC18
HEXIM1
RPS6KB1
SIN3A
HDGF

SMARCC2
WDR6
RPL18
CHD3
PHIP
ACADVL
ISYNA1
MPDZ
CIC
NFIC
DLG4
NBR1
CS
CIRBP
QRICH1
ANKRD52
COL5A2
NAB1
QSER1
NFAT5
PRRC2B
ARID1A
RAP2B
MCL1
CAVIN1
PCDH9
NTS
SYNE2
UBC
VMP1
MACF1
NFIB
ENAH
ZNF217
NR2F2
